# JinHuangJieDu (JHJD) formula attenuates SARS-CoV-2 infection by interrupting RBD-ACE2 binding and HIF-1α-dependent inflammation

**DOI:** 10.3389/fmed.2026.1817137

**Published:** 2026-05-11

**Authors:** Zhan-Qun Yang, Ning Ding, Meng-Zhu Zheng, Jian Wen, Yuan Xue, Li-Ting Zheng, Yi-Heng Yang, Cheng-He Shi, Hua Jiang, Jian Lin, Long Chen

**Affiliations:** 1 Department of Pharmacy, Peking University Third Hospital, Beijing, China; 2 Peking University Third Hospital Cancer Center, Peking University Third Hospital, Beijing, China; 3 Institute of Advanced Clinical Medicine, Peking University, Beijing, China; 4 Department of Traditional Chinese Medicine, Peking University Third Hospital, Beijing, China; 5 Key Laboratory of Tropical Biological Resources of Ministry of Education, School of Pharmaceutical Sciences, Hainan University, Haikou, China; 6 Song Li’s Academician Workstation of Hainan University (School of Pharmaceutical Sciences), Sanya, China; 7 Artificial Auditory Laboratory of Jiangsu Province, Xuzhou Medical University, Xuzhou, China

**Keywords:** COVID-19, HIF-1α, JinHuangJieDu, network pharmacology, RBD

## Abstract

**Background:**

Since the emergence and spread of coronavirus disease 2019 (COVID-19), Traditional Chinese Medicine (TCM) and its formulations have been used extensively in China for preventing and treating COVID-19 with demonstrated clinical efficacy. This study aimed to evaluate both the therapeutic potential and the mechanism of action of a newly designed TCM formula, JinHuangJieDu (JHJD), which is composed of *Lonicerae Japonicae Flos*, *Scutellariae Radix, Pogostemonis Herba*, *Angelicae Dahuricae Radix*, *Taraxaci Herba*, and *Menthae Haplocalycis Herba*.

**Methods:**

The inhibitory effects of JHJD on SARS-CoV-2 spike RBD binding to ACE2 and pseudovirus infection were assessed *in vitro*. Network pharmacology analyses predicted JHJD’s active ingredients, target genes, and enriched pathways. Key targets were validated in vitro for their role in mitigating virus-induced inflammation.

**Results:**

JHJD effectively blocked wildtype and Omicron RBD binding to ACE2 and inhibited pseudovirus infection. Network analyses identified 375 ingredients, 338 targets (primarily in lung, stomach and spleen), and 63 significant pathways related to viral infection/inflammation. Intersectional analysis suggested *CCND1* and *HIF1A* as pivotal genes and *in vitr*o validation confirmed that JHJD targets HIF-1α to attenuate inflammation.

**Conclusion:**

JHJD, a de novo designed TCM formula, effectively inhibits SARS-CoV-2 entry with superior efficacy against Omicron sub-variants. The formulation has a dual mode of action including disruption of the RBD–ACE2 interaction and alleviation of virus-induced inflammation by modulating HIF-1α. These dual mechanisms establish JHJD as a promising therapeutic candidate for COVID-19.

## Graphical Abstract

**Figure:**
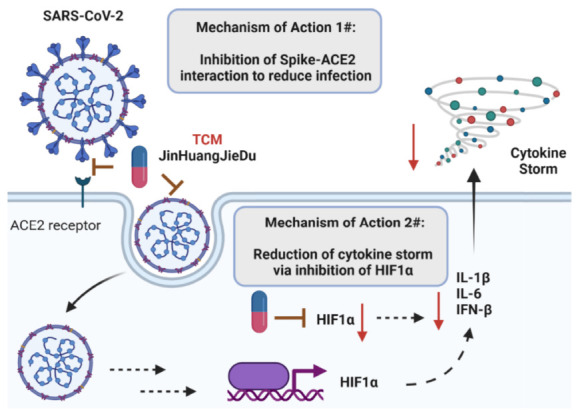
Mechanism of Action of de novo designed TCM JinHuangJieDu. Created in BioRender.Com. This manuscript presents a comprehensive investigation of JinHuangJieDu (JHJD), a de novo designed Traditional Chinese Medicine (TCM) formula rooted in Warm Disease Theory (Wen Bing Xue), demonstrating its dual therapeutic mechanisms against SARS-CoV-2—including Omicron variants—via interruption of RBD-ACE2 binding and modulation of HIF-1α-dependent inflammation. By integrating network pharmacology with experimental validation (pseudovirus neutralization assays, in vitro inflammation models, and transcriptomics), this study bridges classical TCM principles with modern molecular pharmacology and infectious disease research. The work aligns with the scope of Frontiers in Medicine in the following aspects: (1) discovery of multi-target natural product formulations for emerging viral infections; (2) mechanistic insights into host-pathogen interactions and virus-induced inflammatory responses; and (3) evidence-based integration of traditional medicine with contemporary drug discovery strategies. These findings establish JHJD as a promising multi-component therapeutic candidate for COVID-19 and potentially other viral diseases characterized by cytokine storm, offering a compelling contribution to the fields of phytomedicine, antiviral pharmacology, and integrative medicine.

## 1 Introduction

Coronavirus Disease COVID-19, caused by severe acute respiratory syndrome coronavirus 2 (SARS-CoV-2), became a global pandemic since its emergence in December 2019. (1). As of April 30, 2023, over 750 million people have been infected, with 6.8 million overall deaths worldwide (2). SARS-CoV-2 is predominantly transmitted between individuals through droplets (3). For the majority of symptomatic patients, the disease begins with dry cough, fever, dyspnea, myalgia, arthralgia, fatigue, gastrointestinal symptoms, and olfactory/taste disturbances (4, 5). Although the incidence of COVID-19 has been gradually declining, the highly frequent variation of SARS-CoV-2, which may potentially increase its transmissibility, still poses huge threats to public health. Whilst vaccines and a few small molecule drugs have been approved for COVID-19 (6–8), the efficacies of original vaccines for future SARS-CoV-2 variants are in doubt and the efficacy of small molecule drugs still need more evidences (9–11). Therefore, developing new drugs and unveiling the pathogenesis of SARS-CoV-2 infection and COVID-19 are urgently needed.

Traditional Chinese medicine (TCM) has a legacy of millennia and has repeatedly rescued countless lives from epidemics throughout Chinese history. TCM has also provided notable contributions to combating COVID-19 since its emergence in 2019 (12). Studies revealed that certain TCM can significantly reduce the duration of fever and symptom alleviating time in patients with severe COVID-19 (13–15). Meta-analyses demonstrated that combination of Western and Chinese medicine exhibited higher overall efficacy and cure rates, quicker disease remission, lower severe case ratio and thus led to shorter hospital stay compared to Western medicine alone for COVID-19 patients (16). Although traditional Chinese medicine (TCM) exhibits therapeutic potential against SARS-CoV-2 through its multi-component, multi-target mechanisms, the precise underlying actions remain incompletely elucidated.

The receptor-binding domain (RBD) of the SARS-CoV-2 spike (S) protein is the key structure responsible for mediating viral interaction with host cells (17, 18). The S protein is composed of S1 and S2 subunits, with the RBD located within S1 (19). Binding of the RBD to the host angiotensin-converting enzyme 2 (ACE2) receptor enables viral entry into cells (20, 21). Hence, block of S protein RBD interaction with cellular ACE2 is a rational strategy to decrease SARS-CoV-2 infectivity as demonstrated by the promising results using soluble recombinant ACE2 decoys(22) Another characteristic of COVID-19, particularly in severe patients, is excessive inflammation (23), which is highly correlated with disease severity (24).

Herein, we developed a novel TCM formulation, JinHuangJieDu (JHJD), based on the heat-clearing and detoxifying principles of Warm Disease Theory (Wen Bing Xue). It comprises six herbs: *Lonicerae Japonicae Flos* (Jinyinhua, 20g), *Pogostemonis Herba* (Guanghuoxiang, 10g), *Scutellariae Radix* (Huangqin, 10g), *Angelicae Dahuricae Radix* (Baizhi, 10g), *Taraxaci Herb*a (Pugongying, 5g) and *Menthae Haplocalycis Herba* (Bohe, 6g) with a dry weight ratio of 2:1:1:1:1.5:0.6, and is designed to combat COVID-19. Jinyinhua is reused as monarch due to its toxicity clearing properties in blood phase and qi phase internally and externally. Huangqin clears heat and dries dampness, which is good for clearing lung fire, purging heart and stomach fire. Combination of Jinyinhua and Huangqin enhances the ability of clearing heat and removing toxin. Guanghuoxiang dispels dampness to release exterior, harmonizes spleen and stomach, protects stomach and eases middle. Baizhi is pungent and warm and relieves pain and dries dampness. Guanghuoxiang, Huangqin and Baizhi serve as minister. Pugongying clears heat and removes toxin, has strong diuretic effects, cools blood, reduces swelling, disperses knots and enhances the clear heat and remove toxin effects of monarch and returns fire downward. Pugongying serves as assist. Bohe promotes the function of head and eyes, opens skin and hair and induces other Chinese medicine upward. Combination of Bohe, Baizhi and Guanghuoxiang disperses wind, relieves the heat toxin of the upper or surface part of the body and helps Jinyinhua to drive away pathological factors. Bohe serves as envoy. The six herbs together clear heat, remove toxin and dampness and disperse pathogenic factors.

We first proved that JHJD could effectively block SARS-CoV-2 infectivity by inhibiting the interaction between virus RBD and host cell ACE2. This inhibition is effective in different SARS-CoV-2 strains, indicating that JHJD possesses broad-spectrum activity including against SARS-CoV-2 variants. By means of network pharmacology, we identified the key targets of JHJD. In vitro validation demonstrated that JHJD could mitigate the inflammatory responses caused by virus infection through targeting HIF-1α, suggesting that JHJD is a promising drug candidate for COVID-19 with dual anti-infective and anti-inflammatory efficacy.

## 2 Materials and methods

### 2.1 The preparation of JHJD extract

The six herbs in JHJD were mixed in a dry weight ratio of 2: 1: 1: 1: 1.5: 0.6. Then the mixture was decocted and refluxed three times in 12 volumes, 8 volumes and 4 volumes of deionized water for 1 h each. After the extraction, the material was filtered through gauze, and the three aqueous extracts were subsequently combined. Subsequently, the pH was adjusted to 7 by titration with 10% NaOH. The extracts were then placed in 60% ethanol at 4°C for 36 h and the filtrate was adjusted pH to 8 by 10% NaOH titration. After storage at 4°C for 24 h, the JHJD stock solution was filtered, mixed with 0.5% Tween and shaken well, then filtered and autoclaved at 105°C for 30 min to obtain JHJD stock solution with a combined raw drug concentration at 0.284 g/mL (25).

### 2.2 Cell lines and culture conditions

African green monkey SV40-transformed kidney cells (COS7) cells stably expressing hACE2 were prepared by the laboratory (COS7-hACE2) (26). The A549, HEK293T, Hela, and murine macrophage RAW264.7 cells were acquired from ATCC. Specifically, RAW264.7 cells were selected as an *in vitro* model to investigate the initial macrophage-mediated recognition of SARS-CoV-2 and to evaluate the targeted anti-inflammatory properties of the JHJD extract during this crucial phase of infection (27, 28). Cells were maintained in high glucose medium DMEM (Gibco) supplemented with 10% fetal bovine serum (FBS, Gibco) and 100 U/mL penicillin/streptomycin (Gibco). Peripheral blood mononuclear cells (PBMCs) from healthy volunteers were procured from Shanghai Saili Biotechnology Co., Ltd. (China). These cells were cultured in RPMI-1640 medium (Gibco), supplemented with 10% FBS (Gibco), 100 U/mL penicillin-streptomycin, and 100 IU IL2 (GMP-TL906-0050, T&L Biotechnology Co., Ltd., China).

### 2.3 Western blotting

Western blot analysis was performed on HIF-1α and β-actin protein from RAW264.7, COS7-hACE2, Hela, HEK293T and PBMC after drug intervention. Western blotting was performed using anti-HIF-1α antibody (Catalog#: 36169S, Cell Signaling Technology Pathways, United States) and β-actin (Catalog#: 718022, Earthox, United States) at 1:1,000 dilution and HRP-conjugated goat anti-rabbit secondary antibody (Catalog#: E030120-01, Earthox, United States) at 1:1,000 dilution. Images were taken using the GelView 6000 Plus (Guangzhou Biolight Biotechnology Co., Ltd., China).

### 2.4 RT-PCR

Total RNA from the cells was extracted with RNA extraction kit (Catalog#: R1200, Solarbio, Co., Ltd., China) and cDNAs were generated with M-MuLV reverse transcriptase (Catalog#: R1200, Mei5 Biotechnology, Co., Ltd, China). Specific primers and ChamQ SYBR qPCR Master Mix (Catalog#: R1200, Mei5 Biotechnology, Co., Ltd, China) were used for RT-PCR reactions. Each sample was tested in triplicate. Glyceraldehyde-3-phosphate dehydrogenase (GAPDH) was used as a normalization control. The conditions for PCR cycling were: 95° for 30 s, and 40 cycles of 95° for 5 s and 60° for 30 s. All primers used are listed in Supplementary Table 1.

### 2.5 Analysis of SARS-COV2 Spike-ACE2 interaction inhibition using ELISA

S-RBD WT, S-BA.1, S-BA.5, and S-BF.7 proteins (Catalog#: SPD-C522g, Catalog#: SPD-C522r, Catalog#: SPD-C522j Catalog#: SPD-C522y, ACROBiosystems, China) were diluted to a concentration of 1 μg/mL in PBS (Catalog#: P1020, Solarbio, China) and coated on EIA plates overnight at 4°C. Plates were washed once with PBST (0.05% Tween-20). Then, the plates were blocked with 2% BSA in PBS for 2 h at room temperature and washed twice with PBST. JHJD was diluted with PBST-BSA (0.05% Tween and 0.5% BSA in PBS) and pre-incubated in RBD coated, BSA blocked EIA plate for 0.5 h at room temperature. Then, 50 μL of 0.5 μg/mL human ACE2-mFc (Catalog#: AC2-H5205, ACROBiosystems, China) was added and further incubated for 1.5 h at room temperature. The plates were washed 4 times with PBST. 100 μL HRP-conjugated goat anti-mouse secondary antibody (1:5,000 dilution, Catalog#: E030110-01, Earthox, United States) was added per well and incubated for another 1 h at room temperature. Then, the plates were washed 3 times with PBST. TMB solution (100 μL) (Catalog#: PR1200, Solarbio, China) was added to each well and incubated in dark at room temperature. After color development, 50 μL of stop solution (Catalog#: C1058, Solarbio, China) was added to each well to stop the reaction. Absorbance at 450 nm was immediately measured using a multi-well plate reader. Percent of RBD-ACE2 interaction inhibition by JHJD was calculated by normalizing against the PBS-treated control wells, which were assigned a baseline value of 0% inhibition.

### 2.6 SARS-CoV-2 Omicron (BA.5) pseudovirus neutralization

First, to evaluate cell viability, the JHJD extract (stock concentration: 0.284 g/mL) was two-fold serially diluted in culture medium and applied to COS7-hACE2 cells (seeded at a density of 8,000 cells/well). After 24 hours of incubation, cell viability was assessed using a Cell Counting Kit-8 (CCK-8) assay (Catalog#: HY-K0301, MedChemExpress, United States). Subsequently, pseudovirus neutralization was performed based on the previous work with some modifications (26). In brief, SARS-CoV-2 omicron (BA.5) variant pseudo typed lentiviral particles were purchased from BPS Bioscience (Catalog#: 12023ES50, Yeasen, China) with engineered GFP gene to visualize the pseudovirus infection. The anti-hACE2 antibody was used as a reference for the transduction inhibition assay. To validate the neutralization activity of JHJD on BA.5 pseudovirus, COS7-hACE2 cells were seeded in 96-well plates and cultured overnight. JHJD was first diluted with cell culture medium. The lowest dilution factor was 32, resulting in JHJD concentrations of < 5% (v/v). The same volumes of water were added as vehicle controls. The diluted JHJD was pre-incubated with COS7-hACE2 for 30 min before addition of BA.5 pseudovirus. Recombinant ACE2 protein served as positive control. COS7-hACE2 was cocultured with pseudovirus for 1h and then changed to fresh culture medium. After another 48 h culture, cells were harvested and analyzed using flow cytometry by measuring the in cell GFP fluorescence (29). Pseudovirus neutralization by JHJD was determined by the percent of GFP positive cells.

### 2.7 Effects of JHJD on HIF-1α expression and inflammatory factors in different cell lines

To evaluate the impact of JHJD on the expression of HIF-1α and inflammatory mediators, RAW264.7, COS7-hACE2, HeLa, and HEK293T cells were incubated for 24 h with 1.47 μg/mL of JHJD. Subsequently, RAW264.7 cells and PBMCs were stimulated with 50 μg/mL of polyinosinic:polycytidylic acid [Poly(I:C); HY-107202, MCE, United States] to simulate virus-induced inflammation, followed by co-treatment with or without JHJD for an additional 24 h. After the treatment period, a subset of the cells was harvested for total RNA extraction and subsequent q-PCR analysis. The remaining cells were lysed on ice for 30 min using RIPA lysis buffer (R0010, Solarbio, China) supplemented with 1 mM phenylmethylsulfonyl fluoride (PMSF; P0100, Solarbio, China); total protein concentrations were determined using a BCA assay (23225, Thermo, United States) and adjusted to 25 mg/mL. The levels of HIF-1α in the lysates were quantified using ELISA (SEKM-0258, Solarbio, China) and further validated by Western blot analysis.

### 2.8 Network construction and analysis of JHJD targets

A total of 378 chemical constituents and 339 target genes from the six herbs were identified through queries in two databases: the Traditional Chinese Medicine Systems Pharmacology Database and Analysis Platform (TCMSP)^1^ (30) and the Encyclopedia of Chinese Medicine (ETCM)^2^ (31) (Supplementary Table 2). The target gene network was constructed using Cytoscape (version 3.7.1) and analyzed accordingly (32). In the network, nodes represent active ingredients, targets, or pathways. To further visualize the biological functions, gene ontology (GO) enrichment and Kyoto Encyclopedia of Genes and Genomes (KEGG) pathway analysis were performed on these integrated target genes against the standard annotated human genome as the statistical background. This analysis was executed using the ClueGO (version 2.5.8) and CluePedia (version 1.5.8) plug-ins in Cytoscape (33, 34). The top 20 entries with moderate network specificity, a threshold of *p* < 0.05, and the number of enriched genes > 5 were selected for visualization in bubble plots.

### 2.9 Public database analyses

Datasets paired with COVID-19 patients and healthy individuals from the COVID-19 database^3^ were selected and analyzed. For the analysis, three distinct datasets were employed: GSE152641, which comprises 24 healthy controls and 62 patients with community-acquired lower respiratory tract infections due to SARS-CoV-2 within the first 24 h of symptom onset; GSE157103, which includes 26 healthy controls, 60 patients in the intensive care unit (ICU), and 66 non-ICU COVID-19 patients; and GSE165461, which consists of 8 healthy controls, 39 patients with moderate COVID-19, and 25 patients with severe COVID-19. These datasets, representing RNA-Seq data from whole blood, leukocytes, and natural killer (NK) cells, were analyzed to validate the expression of HIF1A in the immune cells of COVID-19 patients.

### 2.10 Protein-protein interaction networks and selection of hub genes

Protein-protein interaction (PPI) analysis of the targets was performed using the STRING database^4^ (35). Proteins of interest were mapped to the PPI network and the interaction score cut-off was set to > 0.4. Then, the PPI network was visualized and constructed using Cytoscape. To identify key PPI network modules, gene network clustering analysis was performed using the MCODE (version 1.6.1) plugin in Cytoscape software with the filtering criteria of k-score = 2, max depth = 100, degree cutoff = 2, node score cutoff = 0.2 (36). CytoHubba (version 0.1) was used to identify important genes in the PPI network as hub genes. The maximum cluster centrality (MCC), maximum neighborhood component (MNC), and edge penetration component (EPC) algorithms were used to identify the top 10 genes (37). Finally, all the results were intersected to obtain the final pivotal gene lists. To correlate the identified hub genes with genes of interest in the transcriptome of COVID-19 patients, covid19db^5^ was used (38), which integrates 9930 drug-target-pathway interactions and 95 COVID-19-related transcriptomic datasets with rich clinical information. Correlation analysis was performed using Pearson correlation.

### 2.11 Statistical analyses

Statistical analysis and graphing of the data were performed using Prism 8.0.1 (Graphpad, La Jolla, CA, United States). Data are expressed as mean ± standard deviation (SD). Statistical significance was determined by one/two-way ANOVA tests after Bonferroni-Hochberg correction. Significance was determined as *p < 0.05, **p < 0.01, ***p < 0.001, ****p < 0.0001.

## 3 Results

### 3.1 Integrated chemical profiling and meridian tropism reveal the multi-herb composition pharmacology of JHJD

**FIGURE 1:**
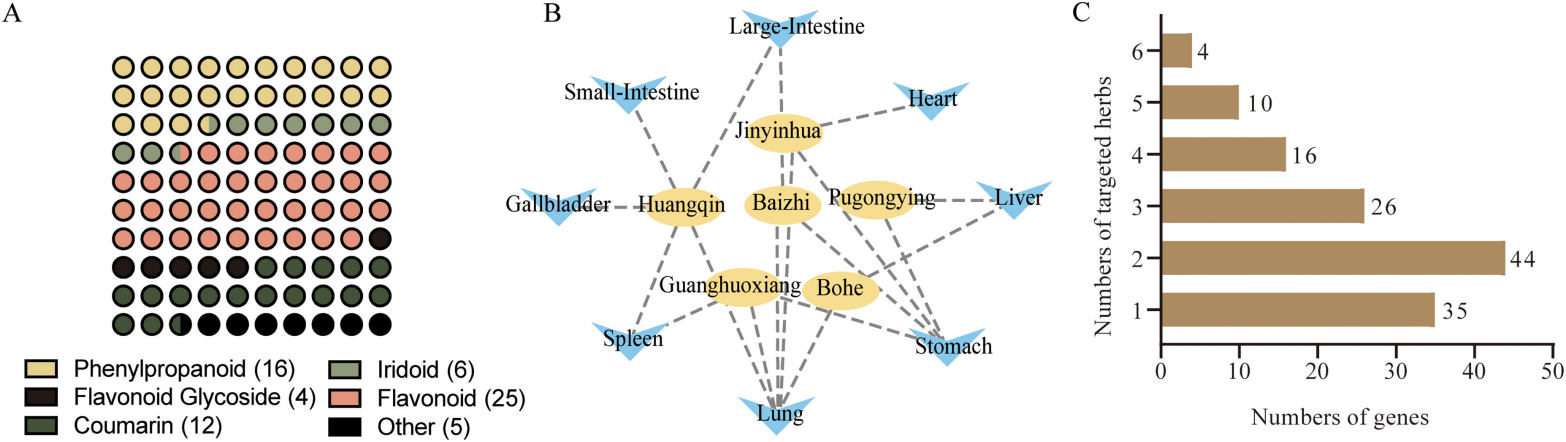
Comprehensive characterization of the composition of JHJD. **(A)** Chemical classification of JHJD components by LC-MS. **(B)** Meridional tropism network of JHJD. Yellow nodes represent the six herbs and blue arrows represent the meridians targeted by individual herbs. **(C)** Numbers of genes targeted by different herbs.

Liquid chromatography–mass spectrometry (LC-MS) profiling of the six-herb formula JHJD (JIN YIN HUA, GUANG HUO XIANG, HUANG QIN, BAI ZHI, PU GONG YING and BO HE) tentatively annotated 68 constituents—16 phenylpropanoids, 6 iridoids, 25 flavonoids, 4 flavonoid glycosides, 12 coumarins and 5 miscellaneous secondary metabolites—through systematic cross-referencing with peer-reviewed literature and public databases (Figure 1A and Supplementary Table 3). Meridional tropism network analysis on the six herbs was conducted, which revealed lung (5) and stomach (4), followed by spleen (2), large intestine (2), and liver (2), heart (1), small intestine (1), and gallbladder (1) (Figure 1B). These results suggest that the herbs may mainly act on the lung, which is the principal organ of SARS-CoV-2 infection. Further analysis revealed a total of 378 active ingredients in JHJD with 339 putative target proteins or genes (Table 1). After removing duplicates, a total of 339 active ingredients and 135 protein or gene targets were identified. In-depth analysis revealed that 35 genes were targeted by one of the six herbs, 44 genes targeted by two herbs, 26 genes targeted by 3 herbs, 14 gene targeted by 4 herbs, 10 genes targeted by 5 herbs and 4 genes (NCOA2, NCOA1, AR, and ESR1) targeted by all of the six herbs (Figure 1C).

**TABLE 1:** Number of compounds contained in herbs of JHJD and corresponding putative targets.

Chinese name	Scientific name	Number of active ingredients	Number of targets
JIN YIN HUA	Lonicerae Japonicae Flos	82	103
GUANG HUO XIANG	Pogostemonis Herba	82	83
HUANG QIN	Scutellariae Radix	84	53
BAI ZHI	Angelicae Dahuricae Radix	84	24
PU GONG YING	Taraxaci Herba	2	25
BO HE	Menthae Haplocalycis Herba	44	51

### 3.2 JHJD broadly inhibits spike RBD-ACE2 interaction

**FIGURE 2:**
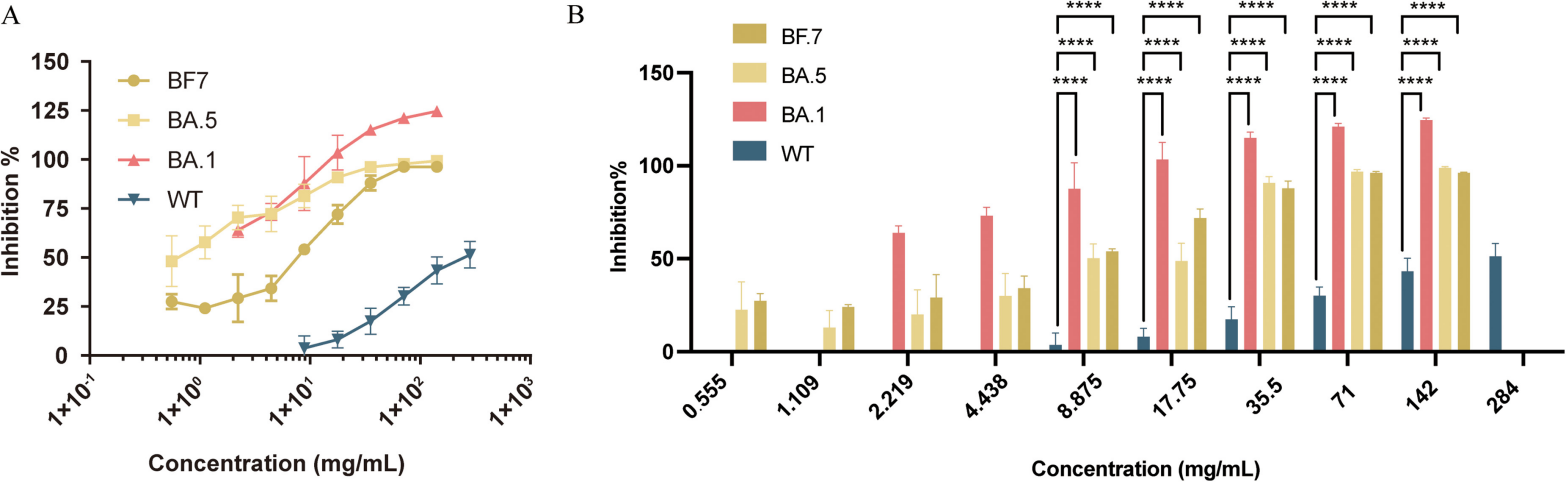
Dose-response curves **(A)** and bar chart **(B)** show JHJD’s inhibition of ACE2 interaction with SARS-CoV-2 WT, BA.1, BA.5 and BF.7 RBD. Data are presented as mean ± SD, PBS was employed as the vehicle control. ****p < 0.0001, two-way ANOVA test, *n* = 5.

SARS-CoV-2 binds to ACE2 with the RBD of the spike protein to facilitate entry into host cells. Therefore, we first examined whether JHJD inhibited the interaction between RBD and ACE2. Notably, using PBS as a control, the inhibition rate was normalized. JHJD inhibited the interaction between the WT RBD and ACE2 in a dose-dependent manner, with the highest inhibition ratio of 51.47 ± 6.78% at a concentration of 284 mg/mL (Figure 2). We further tested the effect of JHJD on three Omicron (B.1.1.529) variants, BA.1, BA.5 and BF.7. JHJD exhibited strong and concentration-dependent inhibition of the interaction of all subvariant RBDs with human ACE2, with inhibition rates approaching 90% at concentrations of 8.875, 35.5 and 35.5 mg/mL for BA.1, BA.5, and BF.7, respectively. Importantly, compared to the WT RBD, JHJD has higher potency for inhibiting interactions of the Omicron variants, BA.5, BF.7 and, particularly, BA.1. For example, at a concentration of 8.875 mg/mL, the inhibition rates of JHJD against BA.5, BF.7, and BA.1 were 13.12, 14.11, and 22.86 times higher than that of the WT RBD, respectively (Figure 2).

### 3.3 JHJD Blocks SARS-CoV-2 entry into host cells

**FIGURE 3:**
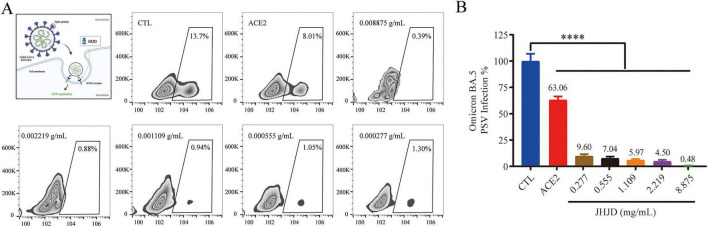
JHJD blocks the entry of Omicron BA.5 pseudovirus into host cells. **(A)** Flow cytometry analysis of Omicron BA.5 spike pseudovirus infection of COS7-hACE2 cells treated with JHJD. A schematic diagram of the pseudovirus-based cellular assay, illustrating the effect of JHJD on SARS-CoV-2 entry into host cells is depicted in the upper left panel. **(B)** Quantitative analysis of the inhibition ratio of JHJD on pseudovirus entry into host cells. CTL = vehicle control (PBS). Data are presented as mean ± SD, *n* = 3. ****p < 0.0001, one-way ANOVA test.

First, we assessed the cytotoxicity of JHJD. The results indicated that cell viability remained at ∼90% at concentrations of 0.002–0.139 mg/mL and dropped slightly to ∼85% at 8.875 mg/mL. However, at a higher concentration of 17.75 mg/mL, cell viability decreased to 75% (Supplementary Figure 1). Next, to determine whether JHJD could effectively block SARS-CoV-2 infectivity, a pseudovirus-based assay was established to evaluate the infectivity of Omicron BA.5 spike pseudo-typed lentivirus harboring GFP in COS7-hACE2 (Figure 3A). An equal volume of PBS served as the blank control, soluble ACE2 protein was used as the positive control, and COS7-hACE2 cells were pre-treated with various concentrations of JHJD. Following a 48-hour incubation period in fresh medium, cellular green fluorescent protein (GFP) fluorescence was quantified using flow cytometry to evaluate the transduction efficiency of the pseudovirus. The results indicated that, in comparison to the PBS control group, the soluble ACE2 positive control group inhibited BA.5 pseudovirus entry by 36.94%. Notably, JHJD treatment demonstrated a robust dose-dependent inhibitory effect on BA.5 pseudovirus entry into ACE2-positive host cells (Figure 3B), achieving an inhibition rate of 90.4% at a concentration of 0.277 mg/mL. Furthermore, at a higher concentration of 8.875 mg/mL, JHJD nearly completely obstructed pseudovirus entry into host cells.

### 3.4 Analysis of JHJD target pathways

**FIGURE 4:**
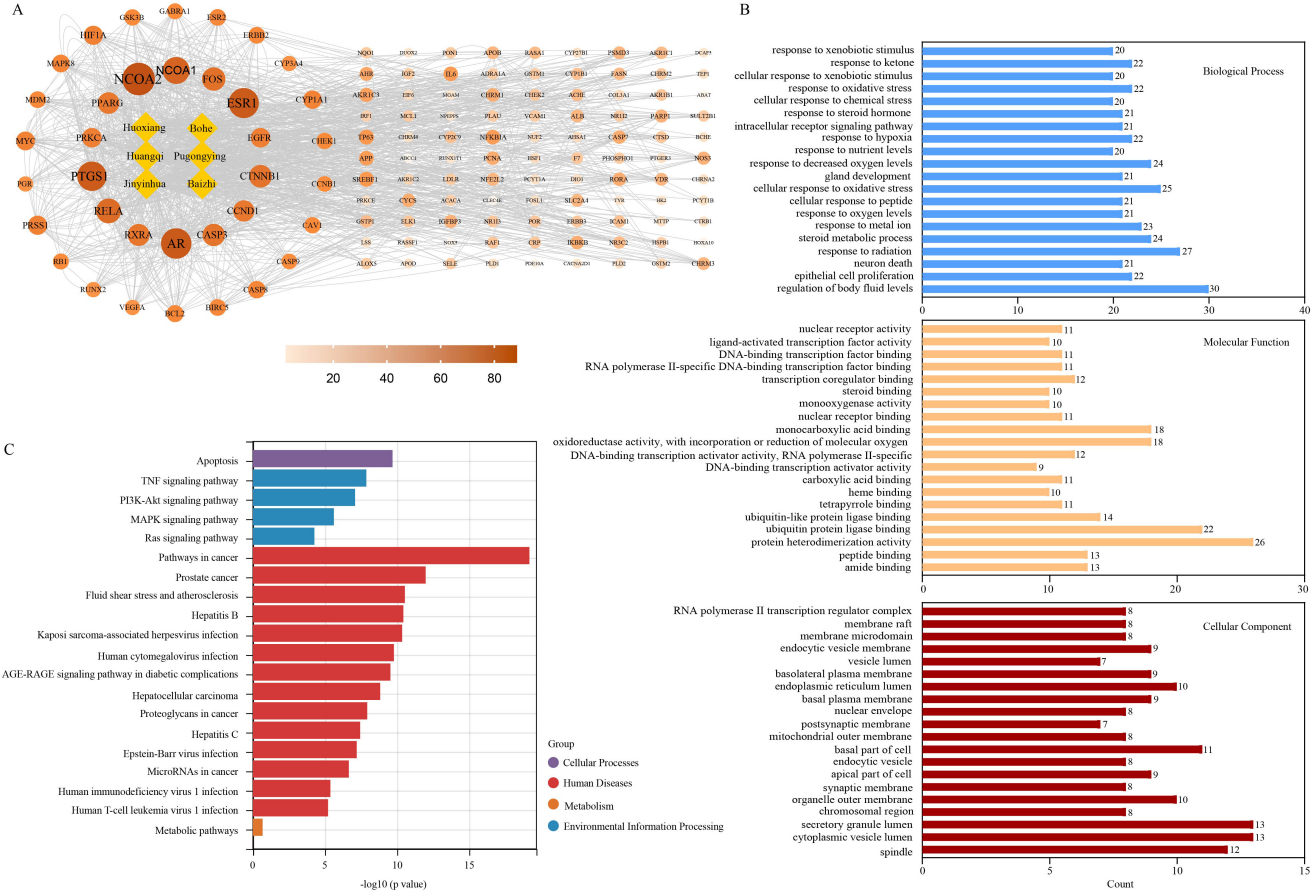
Systems pharmacology network of JHJD. **(A)** PPI network diagram of the effective targets of JHJD. The yellow rectangles represent the six herbs that constitute JHJD. The orange circles represent the effective targets of the six herbs with darker color represent higher degree. Degree of a target was defined as the frequencies of it targeted by the active JHJD ingredients. **(B)** The top 20 pathways of GO enrichment analysis of JHJD. X-axis represents gene numbers in a certain pathway. Blue for biological process, red for cellular component, and yellow for molecular function. **(C)** The top 20 pathways of KEGG enrichment analysis of JHJD. Different colors represent different classification of pathways.

We next performed a comprehensive GO and KEGG enrichment analysis to explore the putative biological footprint of JHJD. After duplicate removal, 135 high-confidence targets were retained (Figure 4A). GO profiling revealed that the top 20 biological-process (BP) terms were dominated by “response to xenobiotic stimulus,” “response to oxidative stress,” “cellular response to chemical stress,” “response to hypoxia/decreased oxygen levels” and “response to steroid hormone,” implying that JHJD may neutralize noxious stimuli, buffer oxidative insults, rescue ischemic injury and modulate endocrine balance. At the cellular-component (CC) level, the target proteins preferentially localize to secretory-granule lumen, cytoplasmic vesicle lumen, endoplasmic-reticulum lumen, basolateral plasma membrane and mitochondrial outer membrane—sites that are pivotal for vesicular trafficking, membrane signaling and apoptotic control. Molecular-function analysis further highlighted prominent enrichment of protein heterodimerization, oxidoreductase, mono-oxygenase, nuclear-receptor, steroid, peptide and ubiquitin(-like) protein ligase binding activities, suggesting that JHJD can remodel redox homeostasis, fine-tune transcriptional complexes and govern protein turnover (Figure 4B). KEGG pathway mining placed “TNF signaling,” “PI3K-Akt signaling,” “MAPK signaling,” “fluid shear stress and atherosclerosis,” “AGE-RAGE signaling in diabetic complications,” “Epstein–Barr virus infection,” “hepatitis B/C,” “HIV-1 infection” and “microRNAs in cancer” among the top 20 cascades (Figure 4C). Collectively, these findings indicate that JHJD targets converge on three core regulatory axes: (i) anti-oxidative and anti-apoptotic cytoprotection, (ii) suppression of pro-inflammatory cytokine networks, and (iii) blockade of viral entry/replication cycles. Consequently, JHJD exhibits a diverse pharmacological profile that may contribute to its anti-inflammatory, antiviral, and metabolic-arterial protective effects.

### 3.5 Search and validation of the key targets of JHJD

**FIGURE 5:**
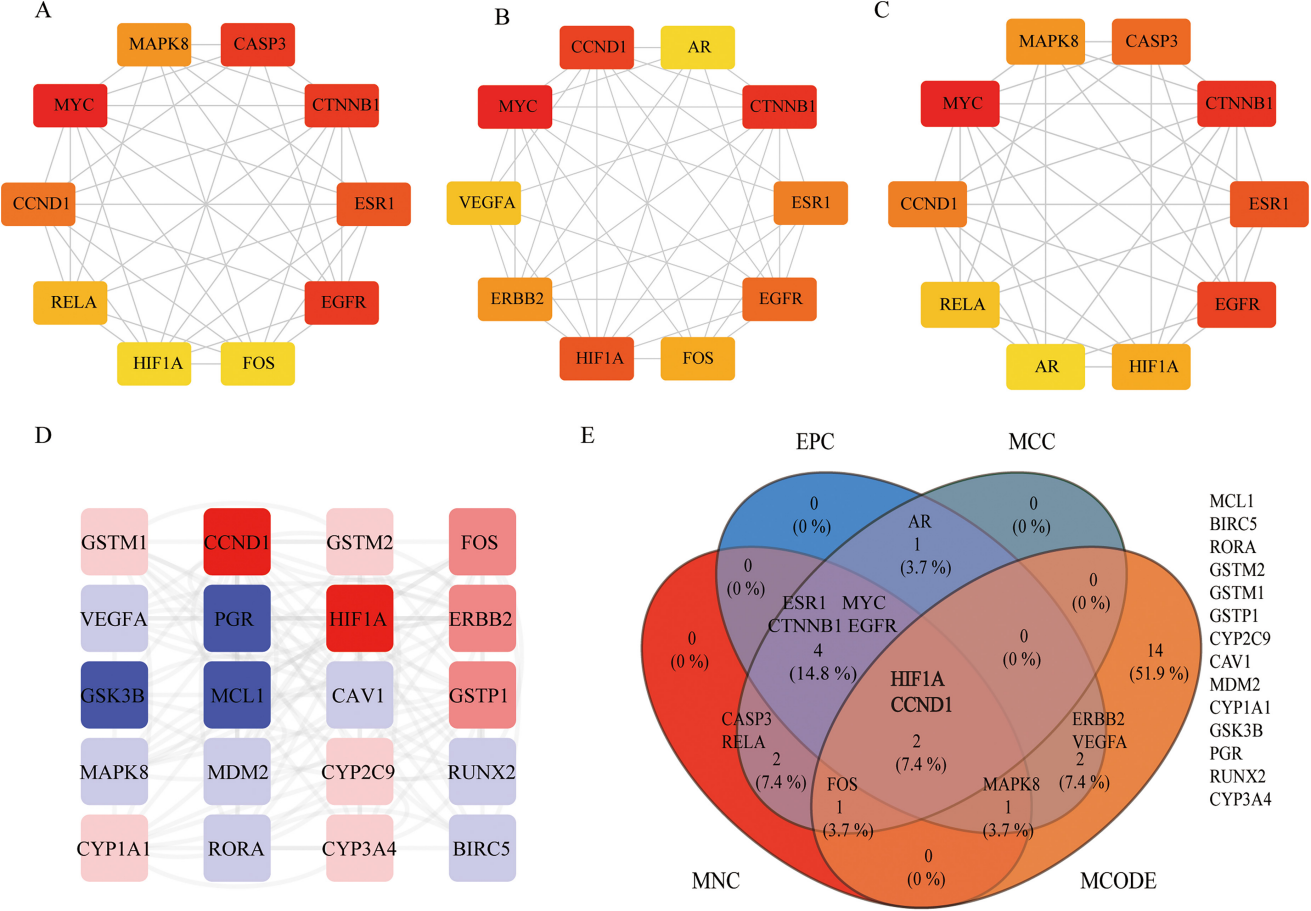
Key targets for JHJD. PPI network of the Top 10 scored proteins obtained using different algorithms: MNC algorithm **(A)**, EPC algorithm **(B)** and MCC algorithm **(C)**. **(D)** Protein targets from the top scored module from MCODE. **(E)** Venn diagram of hub proteins from intersection of key targets from MCC, MNC, EPC, and MOCDE.

Next, protein-protein interactions of the above 135 targets of JHJD were analyzed using STRING. A total of 115 nodes and 996 edges with a composite score > 0.4 were identified and visualized using Cytoscape (Figure 5). Different algorithms were applied to identify the key proteins and the top 10 proteins from each algorithm were selected and displayed (Figures 5A–C). Six key modules were further identified using the MCODE plugin (Figure 5D and Supplementary Table 4, MCODE_Score > 2.5). Intersection of the top 10 targets discovered by the three PPI algorithms and the ones discovered from the top scored MCODE module identified two vital targets: *CCND1* and *HIF1A* (Figure 5E).

**FIGURE 6:**
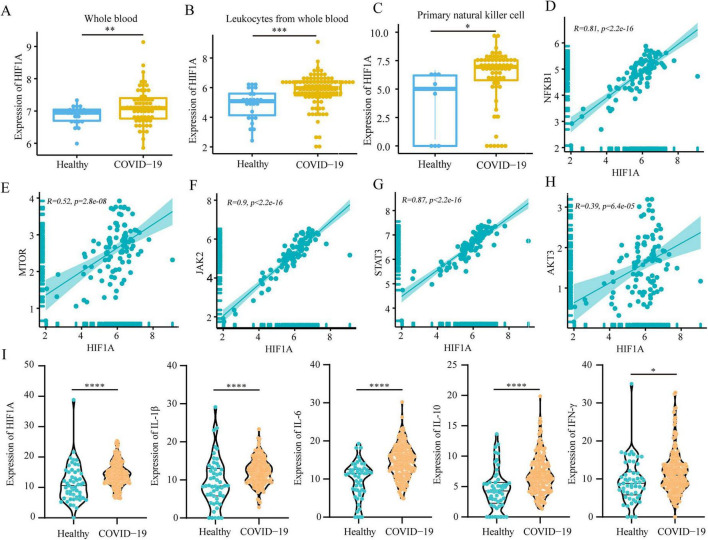
Validation of *HIF1A* expression in COVID-19 patients. **(A–C)** Expression of *HIF1A* in whole blood (**A**, GSE152641), peripheral leukocytes (**B**, GSE157103) and initial natural killer cells (**C**, GSE165461) in COVID-19 patients from available public datasets. **(D–H)** Correlation analysis of *mTOR* **(D)**, *JAK2* **(E)**, *STAT3* **(F)**, *AKT3* **(G)**, *NFKB1* **(H)** with *HIF1A* in peripheral leukocytes (GSE157103) in COVID-19 patients. **(I)** Hydroxymethylation levels of *HIF1A IL-1β*, *IL6*, *IL10* and *IFN-γ* in the plasma cfDNA of COVID-19 patients. Data are presented as mean ± SD. *p < 0.05, **p < 0.01, ***p < 0.001, ****p < 0.0001, one-way ANOVA test. Correlation analysis was performed using Pearson test.

To validate the reliability of the identified targets, three COVID-19-related datasets (including whole blood, peripheral blood lymphocytes and natural killer cells) were retrieved from the GEO database for preliminary verification. Transcriptomic analysis revealed that HIF1A was significantly upregulated in whole blood, peripheral blood lymphocytes and natural killer cells derived from COVID-19 patients (Figures 6A–C), suggesting the involvement of HIF-1α in the pathological progression of COVID-19. COVID-19 is characterized by hypoxia, which leads to activation of PI3K/AKT, mTOR, Jak2/STAT3, NF-kB and inflammasome signaling (39–41). Correlation analyses showed that expression of *HIF1A* is highly correlated with *STAT3*, *NFKB1*, *NFKB2*, *JAK2*, *mTOR* and *AKT3* (Figures 6D–H and Supplementary Figure 2). In addition, hydroxymethylation sequencing data of plasma cfDNA from 53 healthy individuals and 203 patients with COVID-19 (42) also indicated that *HIF1A*, *IL-1β*, *IL6*, *IL10* and *IFN-γ* expression was significantly increased in patients with COVID-19 and that these inflammatory factors correlated with *HIF1A* expression (Figure 6I and Supplementary Figure 3). Thus, we anticipated that JHJD, which targets HIF1A, may also possess another mechanism of action as a therapeutic drug for COVID-19 by mitigating virus-related inflammation through downregulation of HIF1A expression.

### 3.6 JHJD reduces HIF-1α expression and mitigates virus infection-related inflammation *in vitro*

**FIGURE 7:**
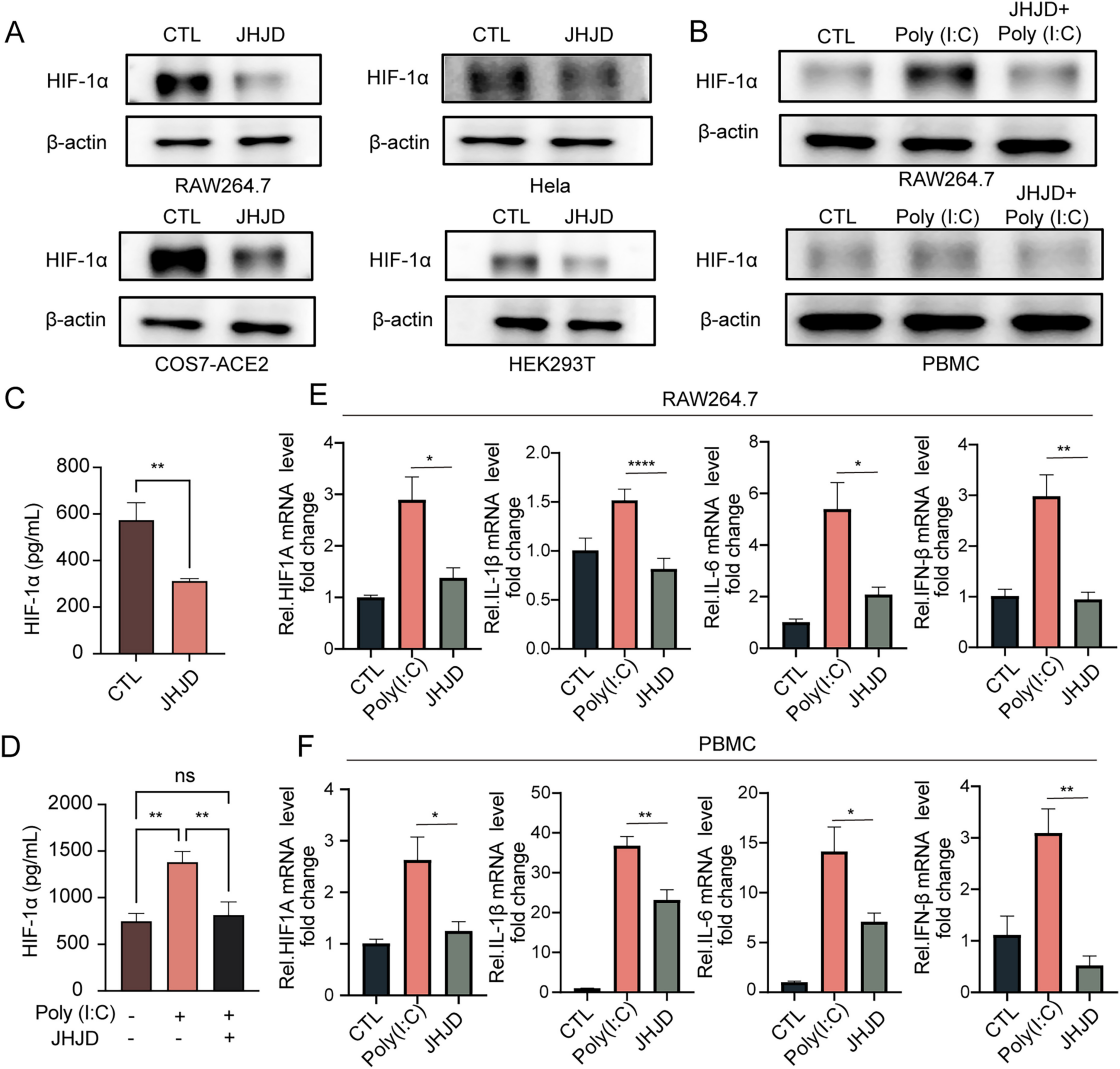
JHJD reduces HIF-1α expression and mitigates inflammation caused by viral infection. **(A)** JHJD reduced HIF-1α expression in RAW264.7, COS7-hACE2, Hela and HEK293T cells. **(B)** JHJD reduced the upregulation of HIF-1α expression in PBMC and RAW264.7 cells induced by Poly(I:C) treatment. **(C)** ELISA quantification of HIF-1α levels in JHJD-treated RAW264.7 cells. **(D)** ELISA quantification of HIF-1α levels in RAW264.7 cells following Poly(I:C) and JHJD treatment. **(E,F)** RT-PCR analysis showing that JHJD reduced *HIF1A*, *IL-1β*, *IL-6* and *IFN-β* transcription that was upregulated by Poly(I:C) treatment in RAW264.7 **(E)** and PBMC **(F)** cells. GAPDH was utilized as the endogenous control and employed for the normalization of data. Data are presented as mean ± SD. *p < 0.05, **p < 0.01, ****p < 0.0001, one-way ANOVA test, *n* = 3.

We anticipated that HIF-1α might play an important role in mitigating inflammation due to viral infection. To this end, we first analyzed if JHJD reduced HIF-1α protein expression in different in vitro models. Importantly, we observed significant reductions in HIF-1α levels in RAW264.7 macrophages as well as in other target cells, including COS7-hACE2, HeLa, and HEK293T (Figure 7A and Supplementary Figures 4–11). To simulate virus infection, RAW264.7 and PBMC cells were treated with Poly(I:C), a dsRNA that mimics RNA virus infection via targeting TLR3 (43). HIF-1α was upregulated with Poly(I:C) and was downregulated upon JHJD treatment (Figure 7B and Supplementary Figures 12–15). In a complementary methodological approach, we conducted an ELISA assay, which corroborated that JHJD effectively reduced HIF-1α levels in RAW264.7 cells, irrespective of the presence or absence of Poly(I:C) (Figures 7C,D). Furthermore, Poly(I:C) induced expression of HIF1A, IL1B, IL6 and IFNB at the mRNA level, which was significantly blunted by JHJD in both RAW264.7 and PBMC cells (Figures 7E,F). Combined, these results demonstrate that JHJD attenuates virus infection-induced inflammation *in vitro*.

## 4 Discussion

COVID-19 has been a global pandemic for several years, causing substantial morbidity and mortality. Although the pandemic gradually comes to an end, the continuous mutation of the virus remains to be a threat to public health. Vaccines and pharmacological interventions are the most effective ways to combat COVID-19. Besides small molecules and therapeutic antibodies, TCM has demonstrated clinical effectiveness in COVID-19, and there remains a pressing need for new TCM preparations. Herein, we present a novel TCM preparation named JinHuangJieDu, on the basis of Traditional Chinese Medicine, aiming to reduce SARS-CoV-2 infectivity.

JHJD was designed de novo according to classical TCM principles to simultaneously block viral entry and dampen excessive inflammation. Using a pseudovirus transduction system, we demonstrated that JHJD prevents interaction between the SARS-CoV-2 spike RBD and ACE2, thereby inhibiting infection by the ancestral strain and the more recent Omicron sub-variants BA.1, BA.5, and BF.7. The enhanced potency against Omicron suggests that JHJD may retain activity against future viral escape mutants. Given that contemporary variants preferentially infect the nasal epithelium (44), reformulating JHJD as a nasal spray or lavage could provide a convenient prophylactic strategy.

Network pharmacology identified 135 putative targets for JHJD; GO and KEGG analyses converged on cancer-related, viral infection, apoptosis, metabolic, TNF, PI3K-Akt and MAPK pathways. Intersection of algorithm-derived gene lists highlighted CCND1 and HIF1A as potential hub targets. While the cell-cycle regulator CCND1 could not be validated, our *in vitro* experiments confirm a direct effect of JHJD on HIF-1α levels and activity. Multiple independent studies have shown that HIF-1α enhances viral entry and replication while amplifying cytokine release (45–48). Consistent with these reports, we confirmed up-regulation of HIF1A in the circulating leukocytes of COVID-19 patients. The nuclear accumulation of HIF-1α in immune cells is likely facilitated by prolyl hydroxylase domain (PHD) -dependent hydroxylation and by direct transcriptional activation via NF-κB, STAT3 and TLR4-mTOR pathways. The resulting metabolic reprogramming toward glycolysis exacerbates tissue hypoxia and fuels a self-perpetuating inflammatory loop (45–47). Our data indicate that JHJD suppresses HIF-1α expression in vitro and attenuates virus-induced cytokine elevations, supporting the hypothesis that HIF-1α inhibition is a key mechanism by which JHJD mitigates generalized viral hyperinflammation, which likely contributes to its overall efficacy against COVID-19-associated cytokine storms.

We acknowledge several limitations. First, the use of pseudoviruses—dictated by biosafety considerations—precludes direct assessment of JHJD’s impact on authentic viral replication kinetics. Second, although we have shown that JHJD down-regulates HIF-1α, the precise molecular interactions underlying this effect remain to be fully elucidated. Third, while the Poly(I:C) model has been employed in previous COVID-19 research to mimic virus-induced inflammatory responses, it remains a generalized model of viral inflammation with limited specificity for SARS-CoV-2. Given the constraints of current experimental conditions, future studies should focus on developing authentic virus-based inflammatory models to enhance pathophysiological relevance. Therefore, while our data clearly demonstrate JHJD’s modulatory effect on the general HIF-1α-linked inflammatory axis, definitively confirming SARS-CoV-2-unique pathophysiological mechanisms requires further investigation using authentic live viruses. Finally, preliminary LC-MS/MS and docking analyses suggest that luteolin and quercetin—constituents of the herbal components within JHJD—may occupy the HIF-1α binding pocket and destabilize the protein, but these predictions require rigorous biochemical confirmation.

Notwithstanding these caveats, JHJD exemplifies a modern, evidence-based application of TCM for infectious disease control. By simultaneously blocking viral entry and attenuating HIF-1α-driven inflammation, JHJD offers a dual-mechanism antiviral approach that could extend beyond COVID-19 to other viral infections characterized by excessive inflammatory responses.

## 5 Conclusion

De-novo TCM formula JHJD inhibits SARS-CoV-2 especially Omicron escape variants by blocking RBD–ACE2 engagement and attenuates generalized virus-induced, HIF-1α-driven inflammation, potentially mitigating COVID-19 severity. These findings provide a theoretical basis and methodological reference for further development and utilization of JHJD in COVID-19 and emphasized the critical roles of TCM in treating emerging infectious diseases.

## Data Availability

The original contributions presented in the study are included in the article/Supplementary material, further inquiries can be directed to the corresponding author.

## Footnotes

1
https://tcmsp-e.com/tcmsp.php

2
http://www.nrc.ac.cn:9090/ETCM/

3
www.covid19db

4
https://string-db.org/

5
http://www.biomedical-web.com/covid19db
