# Supplementary material for: JinHuangJieDu (JHJD) formula attenuates SARS-CoV-2 infection by interrupting RBD-ACE2 binding and HIF-1α-dependent inflammation
Source: Front Med (Lausanne). 2026 May 11;13:1817137. doi: 10.3389/fmed.2026.1817137 (PMC13199254; doi:10.3389/fmed.2026.1817137)
Supplement: Supplementary file 1 [file Supplementary_file_1.docx]

**Supporting Information**

Supplementary table 1 Primers used in this study.

| Human *GAPDH* Forward:  5’-CGGAGTCAACGGATTTGGTC-3’ | Human *GAPDH* Reverse:  5’-GACAAGCTTCCCGTTCTCAG-3’ |
| --- | --- |
| Human *HIF1A* Forward:  5’-CGTCGAAAAGAAAAGTCTCGAGAT-3’ | Human *HIF1A* Reverse:  5’-AGGCCTTATCAAGATGCGA ACT-3’ |
| Human *IL-1β* Forward:  5’-CTCTCTCCTTTCAGGGCCAA-3’ | Human *IL-1β* Reverse:  5’-GAGAGGCCTGGCTCAACAAA-3’ |
| Human *IL-6* Forward:  5'-AGACAGCCACTCACCTCTTCAG-3' | Human *IL-6* Reverse:  5'-TTCTGCCAGTGCCTCTTTGCTG-3' |
| Human *IFN-β* Forward:  5’-ATTGCCTCAAGGACAGGAG-3’ | Human *IFN-β* Reverse:  5’-GGCCTTCAGGTAATGCAGAA-3’ |
| Mouse *GAPDH* Forward:  AAGCCCATCACCATCTTCCA | Mouse *GAPDH* Reverse:  CCTGCCTCACCACCTTCTTG |
| Mouse *HIF1A* Forward:  GACAGAGCCGGCGTTTAGG | Mouse *HIF1A* Reverse:  CGACGTTCAGAACTCATCCTATTTT |
| Mouse *IL-1β* Forward:  AGGACATGAGCACCTTCTTTTCC | Mouse *IL-1β* Reverse:  CGACGTTCAGAACTCATCCTATTTT |
| Mouse *IL-6* Forward:  TCGGAGGCTTAATTACACATGTTC | Mouse *IL-6* Reverse:  CATACAATCAGAATTGCCATTGC |
| Mouse *IFNβ* Forward:  CCATCATGAACAACAGGTGGAT | Mouse *IFNβ* Reverse:  GAGAGGGCTGTGGTGGAGAA |

Supplementary table 3 Chemical composition of JHJD analyzed by LC-MS.

| NO. | RT  (min) | Identity | Mass charge ratio  (m/z) | Molecular  fomula | Error (ppm) | Type |
| --- | --- | --- | --- | --- | --- | --- |
| 1 | 3.4 | Cryptochlorogenic acid | 353.08781 | C16H18O9 | 0.5 | Phenylpropanoid |
| 2 | 3.43 | Caffeic acid | 179.03498 | C9H8O4 | 0.1 | Phenylpropanoid |
| 3 | 3.57 | Secologanic acid | 373.11402 | C16H22O10 | -1.4 | Iridoid |
| 4 | 4.19 | Secologanoside 7-methyl ester | 389.10894 | C16H22O11 | -1 | Iridoid |
| 5 | 4.45 | Chlorogenic acid | 353.08746 | C16H18O9 | -1 | Phenylpropanoid |
| 6 | 5.06 | Sweroside | 358.126383 | C16H22O9 | -3.4 | Iridoid |
| 7 | 5.06 | secoxyloganin | 403.12459 | C17H24O11 | -1.2 | Iridoid |
| 8 | 5.07 | LONICERIN | 593.15119 | C27H30O15 | -2.2 | Flavonoid |
| 9 | 7.1 | Rutin | 609.14611 | C27H30O16 | -2.5 | Flavonoid |
| 10 | 7.27 | Ellagic acid | 300.99899 | C14H6O8 | -0.8 | Tannin |
| 11 | 7.56 | Cynaroside | 447.09329 | C21H20O11 | -2 | Flavonoid |
| 12 | 7.59 | ISOQUERCITRIN | 463.0882 | C21H20O12 | -3.7 | Flavonoid |
| 13 | 7.64 | Ferulic Acid | 193.05063 | C10H10O4 | -0.9 | Phenylpropanoid |
| 14 | 7.64 | Luteolin | 285.04046 | C15H10O6 | -2.3 | Flavonoid |
| 15 | 8.22 | Isochlorogenic Acid B | 515.1195 | C25H24O12 | 0.3 | Phenylpropanoid |
| 16 | 8.71 | Isochlorogenic acid C | 516.12678 | C25H24O12 | 1 | Phenylpropanoid |
| 17 | 9.38 | Isochlorogenic acid A | 515.1195 | C25H24O12 | -1.8 | Phenylpropanoid |
| 18 | 10.38 | Quercetin | 301.03538 | C15H10O7 | -4.3 | Flavonoid |
| 19 | 12.49 | Kaempferol | 285.04046 | C15H10O6 | -4.5 | Flavonoid |
| 20 | 14.19 | macranthoidin A | 1235.60662 | C59H96O27 | -1.7 | Iridoid |
| 21 | 24.64 | Hederagenin | 471.34798 | C30H48O4 | -8.8 | Iridoid |
| 22 | 5.07 | VICENIN | 593.15119 | C27H30O15 | -2.2 | Flavonoid |
| 23 | 6.35 | Campneoside II | 639.19306 | C29H36O16 | -2.9 | Phenylpropanoid |
| 24 | 8.11 | Verbascoside | 623.19588 | C29H36O15 | -3.6 | Phenylpropanoid |
| 25 | 8.95 | Azelaic acid | 187.09758 | C9H16O4 | -1.2 | Other |
| 26 | 9.65 | [(1R,6S,7S,8R,9R)-4-(3,4-dihydroxyphenyl)-9-(hydroxymethyl)-7-[(2S,3R,4R,5S,6S)-3,4,5-trihydroxy-6-methyl-oxan-2-yl]oxy-2,5,10-trioxabicyclo[4.4.0]dec-8-yl] (E)-3-(3,4-dihydroxyphenyl)prop-2-enoate | 621.18249 | C29H34O15 | -2.3 | Phenylpropanoid |
| 27 | 10.5 | Apigenin | 269.04549 | C15H10O5 | -0.2 | Flavonoid |
| 28 | 10.5 | APIGENIN-7-GLUCURONIDE | 445.07764 | C21H18O11 | -0.2 | Flavonoid Glycoside |
| 29 | 10.82 | DHELWANGIN | 223.09758 | C12H16O4 | -3.8 | Other |
| 30 | 11.69 | Linarin | 591.17193 | C28H32O14 | -3.1 | Flavonoid Glycoside |
| 31 | 12.12 | QUERCETIN-3,7,3',4'-TETRAMETHYL ETHER | 357.09798 | C19H18O7 | -12.9 | Flavonoid |
| 32 | 14.63 | Diosmetin | 299.05611 | C16H12O6 | -3.2 | Flavonoid |
| 33 | 16.1 | 4',7-DIMETHOXY-3,3',5-TRIHYDROXYFLAVONE | 329.06668 | C17H14O7 | -3.3 | Flavonoid |
| 34 | 19.23 | 4',5-DIHYDROXY-7-METHOXYFLAVONE | 283.0612 | C16H12O5 | -2.3 | Flavonoid |
| 35 | 19.37 | 7,4'-DI-O-METHYLERIODICTYOL | 315.08741 | C17H16O6 | -4.8 | Flavonoid |
| 36 | 19.43 | irisolidone | 313.07176 | C17H14O6 | -2.4 | Flavonoid |
| 37 | 21.18 | pachypodol | 343.08233 | C18H16O7 | -3.1 | Flavonoid |
| 38 | 3.35 | N-[(tert-Butoxy)carbonyl]-D-tryptophan | 203.0826 | C_11_H_12_N_2_O_2_ | -1.9 | Other |
| 39 | 5.68 | 4-Hydroxy-3,5-dimethoxycinnamic acid | 223.0612 | C_11_H_12_O_5_ | -0.5 | Phenylpropanoid |
| 40 | 10.38 | 6-hydroxyluteolin | 301.03538 | C_15_H_10_O_7_ | -4.3 | Flavonoid |
| 41 | 10.5 | Baicalein | 269.04555 | C_15_H_10_O_5_ | -0.2 | Flavonoid |
| 42 | 10.5 | Baicalin | 445.07764 | C_21_H_18_O_11_ | -0.2 | Flavonoid Glycoside |
| 43 | 12.6 | (E)-3-(4-hydroxy-3-methoxy-phenyl)acrylic acid methyl este | 207.06628 | C_11_H_12_O_4_ | -1.6 | Phenylpropanoid |
| 44 | 12.98 | Beta-D-Glucopyranosiduroic acid | 459.09329 | C_22_H_20_O_11_ | -1.8 | Glycoside |
| 45 | 14.63 | 5,7,4'-trihydroxy-8-methoxyflavone | 299.05611 | C_16_H_12_O_6_ | -3.2 | Flavonoid |
| 46 | 16.1 | Viscidulin II | 329.06668 | C_17_H_14_O_7_ | -3.3 | Flavonoid |
| 47 | 19.23 | Wogonin | 283.0612 | C_16_H_12_O_5_ | -2.3 | Flavonoid |
| 48 | 19.43 | Rivularin | 313.07176 | C_17_H_14_O_6_ | -2.4 | Flavonoid |
| 49 | 19.83 | Pinocembrine | 255.06628 | C_15_H_12_O_4_ | -7.6 | Flavonoid |
| 50 | 20.66 | 5-hydroxy-7,8-dimethoxyflavone | 297.07685 | C_17_H_14_O_5_ | -5.5 | Flavonoid |
| 51 | 20.75 | Naringenin | 271.0612 | C_15_H_12_O_5_ | -3.4 | Flavonoid |
| 52 | 3.75 | Oxypeucedaninhydrate | 303.08741 | 303.08741 | 4 | Coumarin |
| 53 | 6.16 | Byakangelicol | 631.1821 | 631.1821 | -0.8 | Coumarin |
| 54 | 6.88 | Skimmin | 323.07724 | 323.07724 | -3.6 | Coumarin |
| 55 | 7.67 | Umbelliferone | 161.02442 | 161.02442 | 0.1 | Coumarin |
| 56 | 11.65 | Xanthotoxol | 201.01933 | 201.01933 | -2.3 | Coumarin |
| 57 | 13.29 | Oxypeucedanin | 285.07685 | 285.07685 | -2.9 | Coumarin |
| 58 | 15.3 | Osthole | 243.10267 | 243.10267 | -2.4 | Coumarin |
| 59 | 15.3 | Suberosin | 243.10267 | 243.10267 | -2.4 | Coumarin |
| 60 | 20.25 | osthenol | 229.08702 | 229.08702 | -2.5 | Coumarin |
| 61 | 20.51 | Imperatorin | 269.08116 | 269.08116 | -2.9 | Coumarin |
| 62 | 20.85 | Isoimperatorin | 269.08193 | 269.08193 | -2.4 | Coumarin |
| 63 | 20.85 | Alloimperatorin | 269.08193 | 269.08193 | -2.4 | Coumarin |
| 64 | 2.76 | Vanillic acid | Vanillic acid | 167.03498 | -0.2 | Phenylpropanoid |
| 65 | 3.44 | Caftaric Acid | Caftaric Acid | 311.04086 | -2 | Phenylpropanoid |
| 66 | 5.69 | Syringic acid | Syringic acid | 197.04555 | -1.8 | Phenylpropanoid |
| 67 | 7.43 | ChicoricAcid | ChicoricAcid | 473.07255 | -0.4 | Phenylpropanoid |
| 68 | 17.11 | hesperetin | hesperetin | 301.07176 | -1.2 | Flavonoid |

Supplementary table 4 Six models with MCODE scores > 2.5

| Cluster | Score | Nodes | Edges | Node IDs |
| --- | --- | --- | --- | --- |
| 1 | 5.263 | 20 | 100 | *MCL1, BIRC5, ERBB2, RORA, GSTM2, GSTM1, VEGFA, GSTP1, FOS, CYP2C9, CAV1, MDM2, CYP1A1, GSK3B, PGR, MAPK8, CCND1, RUNX2, CYP3A4, HIF1A* |
| 2 | 4 | 4 | 12 | *ESR1, CTNNB1, MYC, EGFR* |
| 3 | 3.6 | 6 | 18 | *RB1, CASP9, CCNB1, CASP3, HSPB1, CYCS* |
| 4 | 3 | 3 | 6 | *PCYT1A, PCYT1B, PHOSPHO1* |
| 5 | 3 | 3 | 6 | *NR1I2, RXRA, NCOA1* |
| 6 | 2.545 | 12 | 28 | *CRP, VCAM1, IKBKB, SELE, NOS3, ERBB3, AR, ESR2, RELA, ALB, NFKBIA, NCOA2* |


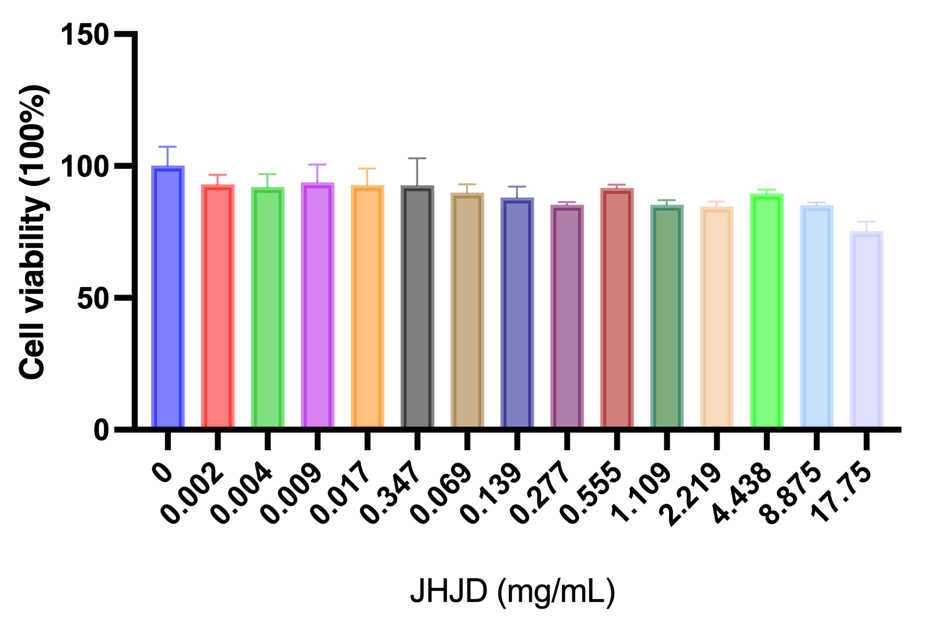


Supplementary figure 1. Effect of various JHJD concentrations on the viability of COS7-ACE2 cells after 24 hours of treatment. n=3.
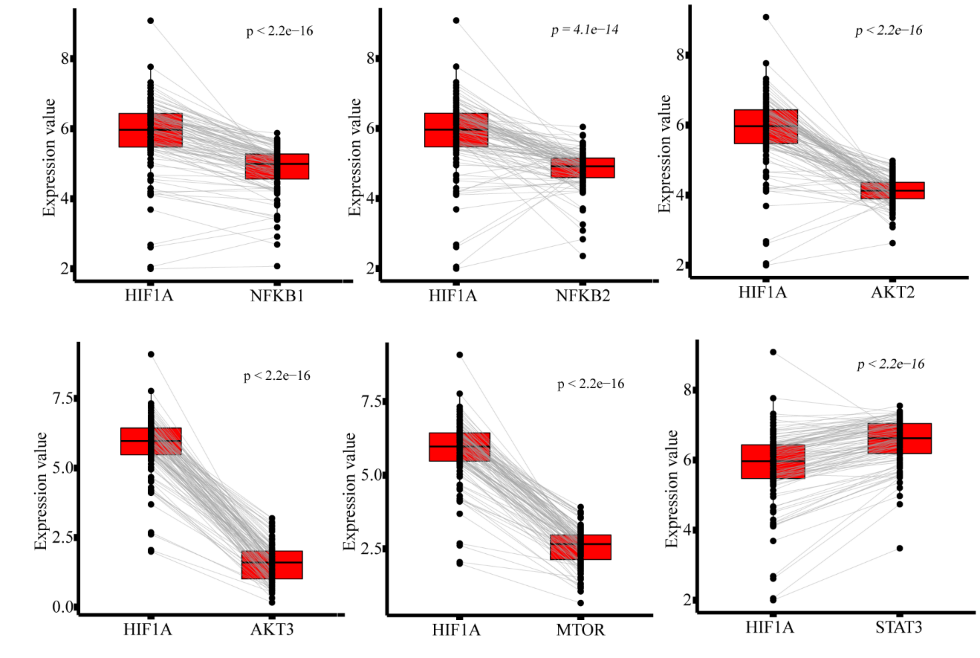


Supplementary figure 2. Box plots of *mTOR*, *JAK2*, *STAT3*, *AKT3*, *NFKB1*, *NFKB2* and *HIF1A* gene expression levels in plasma leukocytes (GSE157103) of COVID-19 patients. The t-test was used for comparison between the two groups.
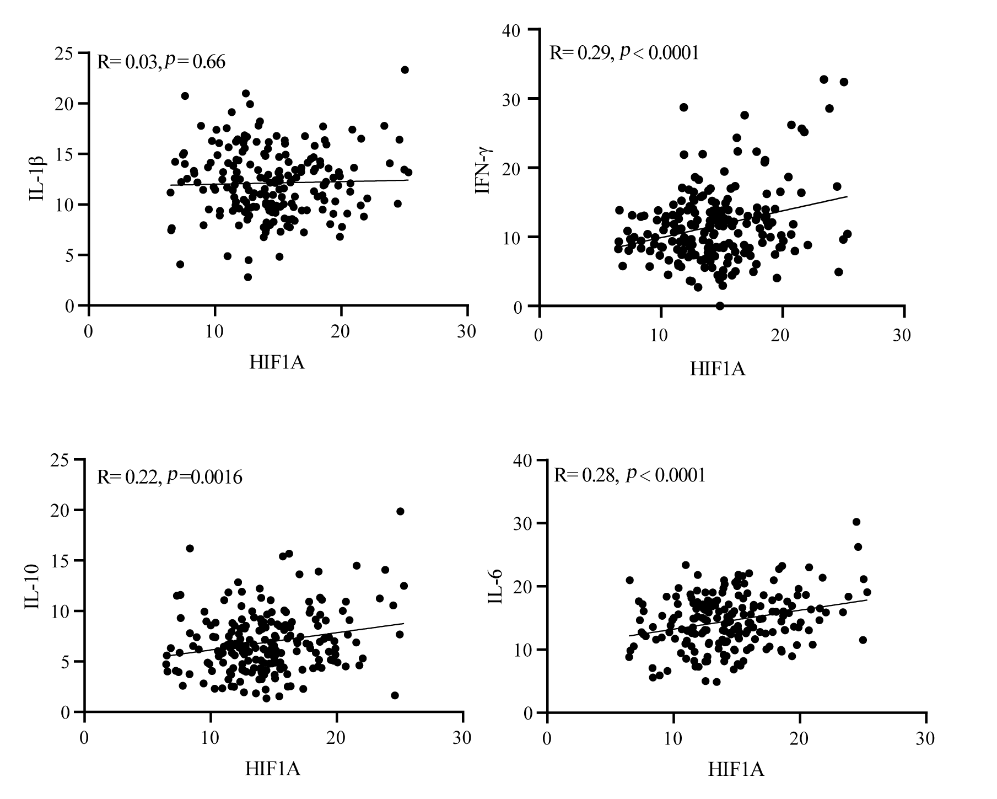


Supplementary figure 3. Correlation analysis of plasma levels of *IL1-β*, *IFN-γ*, *IL-10* and *IL-6* with *HIF1A* hydroxymethylation in COVID-19 patients.


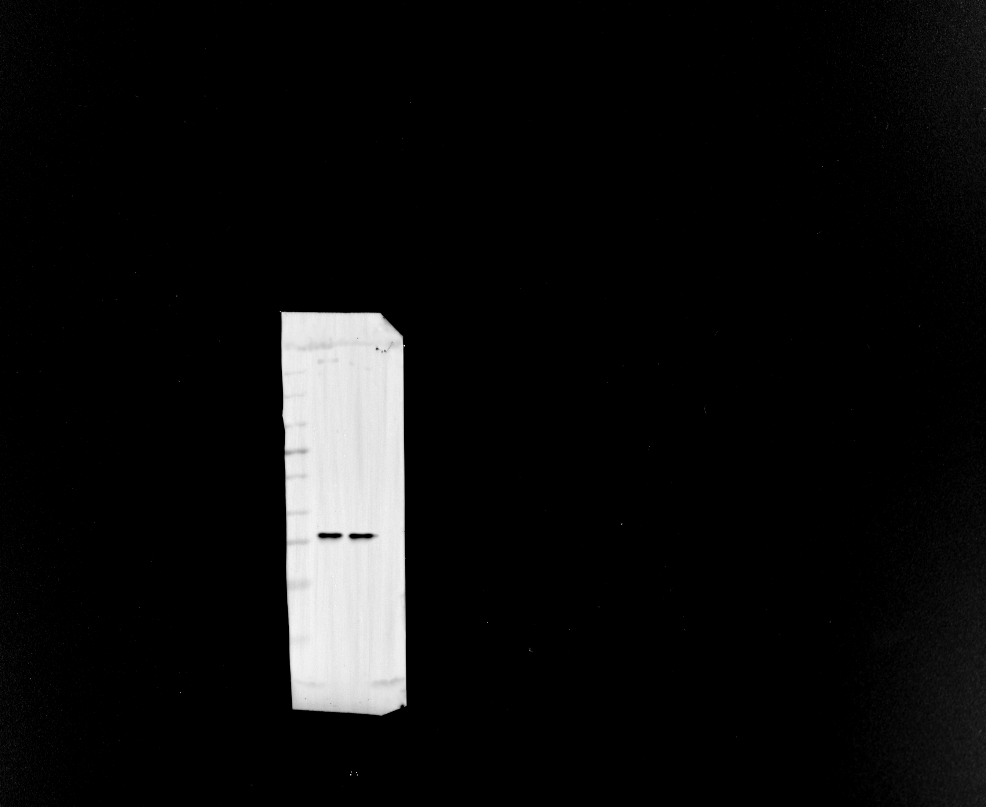


180 kd

135 kd

100 kd

75 kd

65 kd

45 kd

35 kd

25 kd

15 kd

10 kd

GAPDH

Supplementary figure 4. Expression of GAPDH in RAW264.7 cells following treatment with JHJD. Related to Figure 7A.


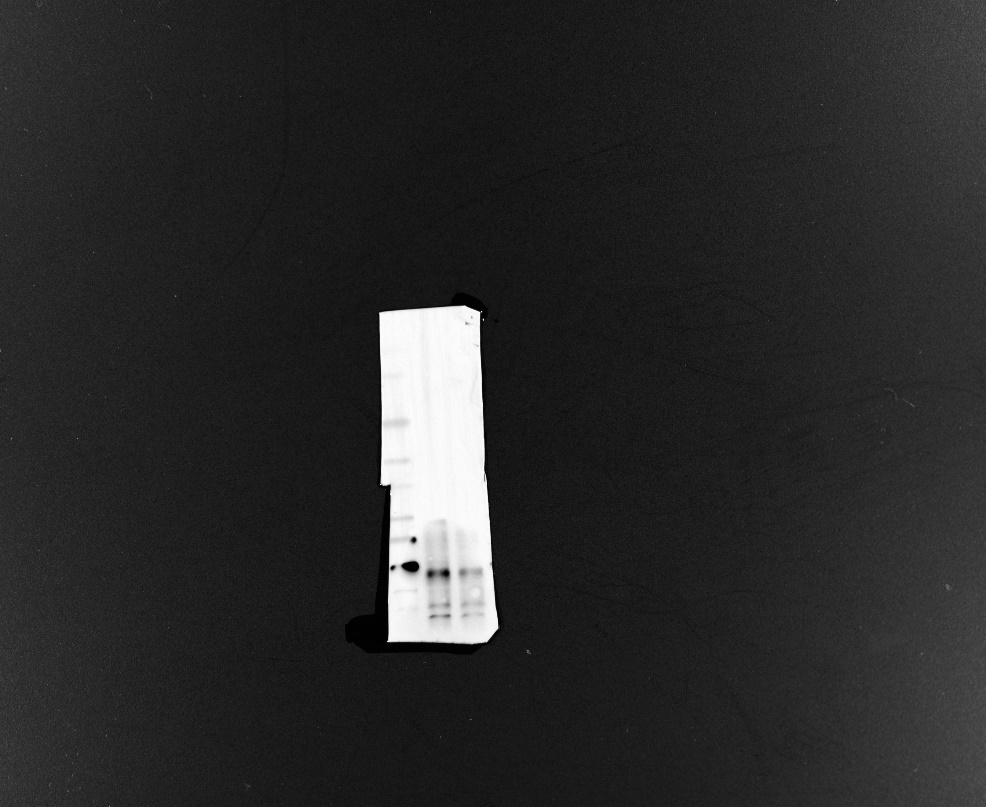


HIF-1α

135 kd

180 kd

180 kd

100 kd

75 kd

65 kd

45 kd

35 kd

25 kd

15 kd

Supplementary figure 5. Expression of HIF-1α in RAW264.7 cells following treatment with JHJD. Related to Figure 7A.


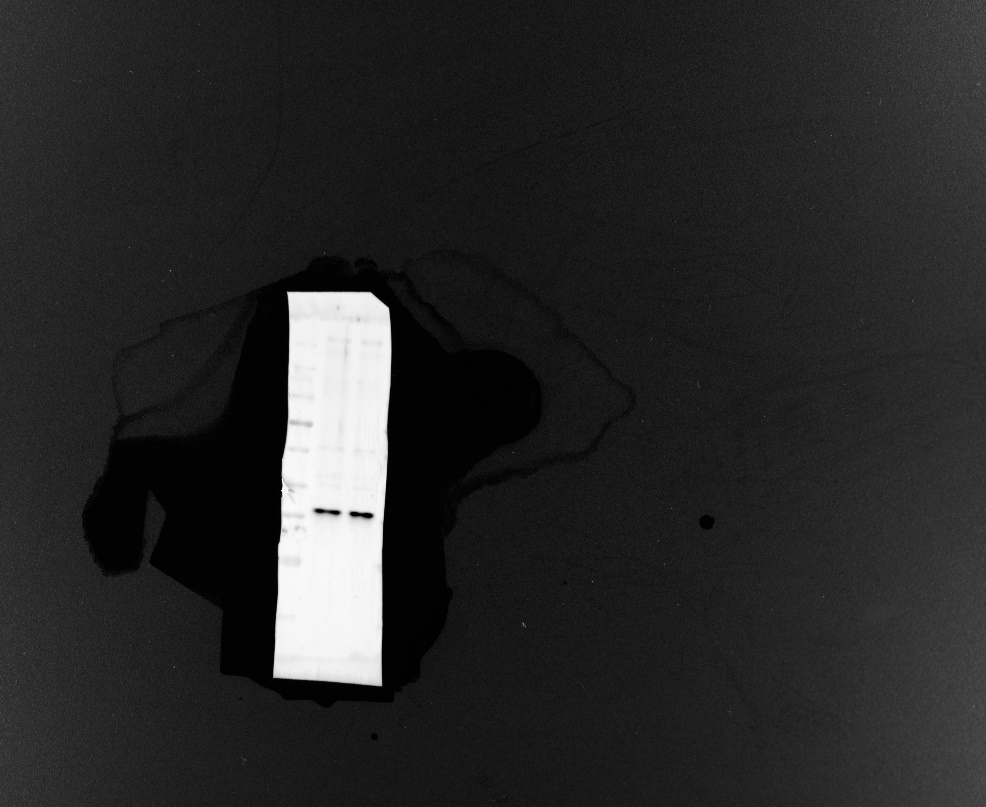


GAPDH

180 kd

135 kd

100 kd

75 kd

65 kd

45 kd

35 kd

25 kd

15 kd

Supplementary figure 6. Expression of GAPDH in Hela cells following treatment with JHJD. Related to Figure 7A.


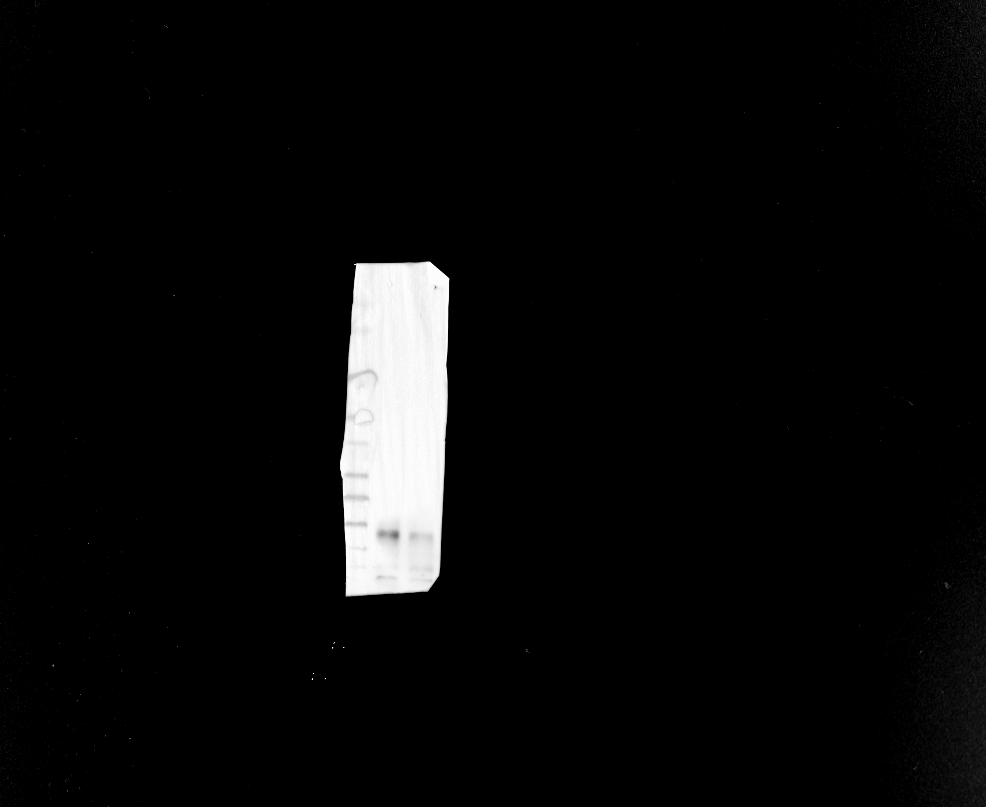


HIF-1α

180 kd

135 kd

100 kd

75 kd

65 kd

45 kd

35 kd

25 kd

10 kd

15 kd

Supplementary figure 7. Expression of HIF-1α in Hela cells following treatment with JHJD. Related to Figure 7A.


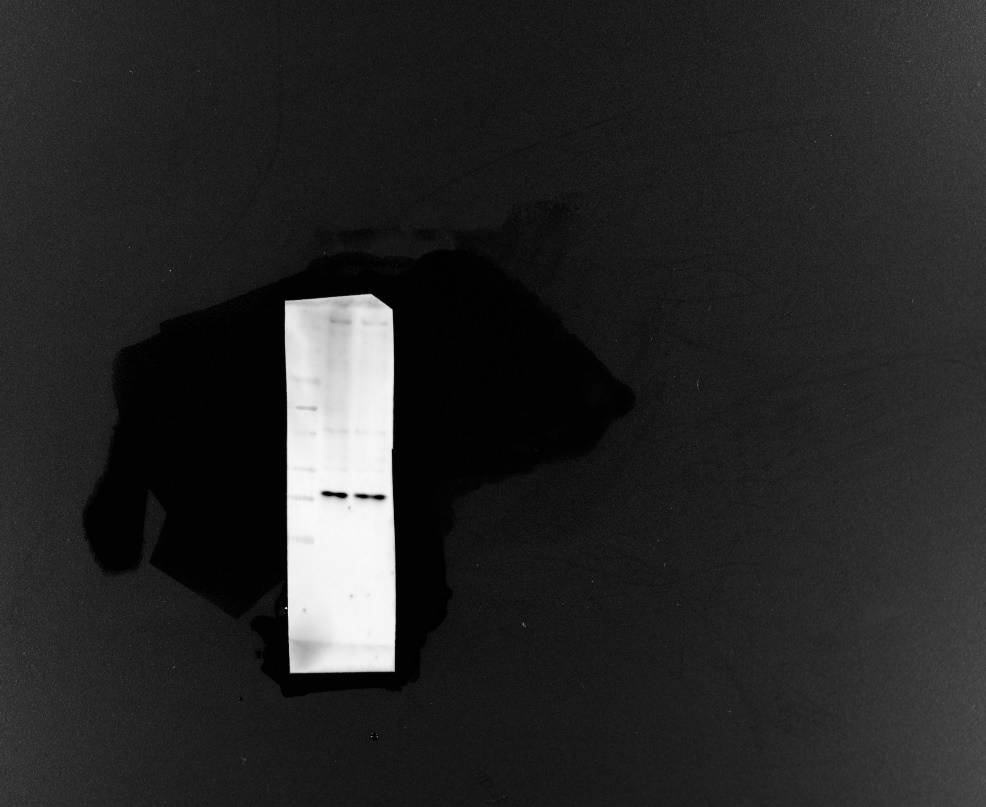


GAPDH

180 kd

135 kd

100 kd

75 kd

65 kd

45 kd

35 kd

25 kd

Supplementary figure 8. Expression of GAPDH in 293T cells following treatment with JHJD. Related to Figure 7A.


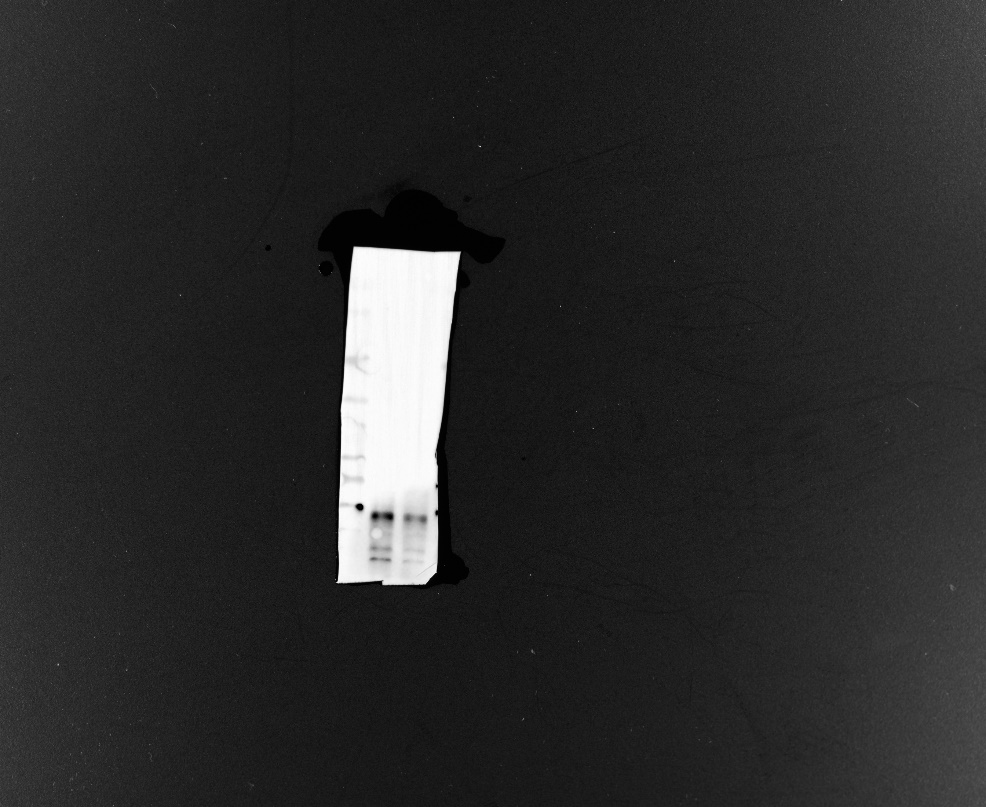


HIF-1α

135 kd

100 kd

75 kd

65 kd

45 kd

35 kd

25 kd

15 kd

Supplementary figure 9. Expression of HIF-1α in 293T cells following treatment with JHJD. Related to Figure 7A.


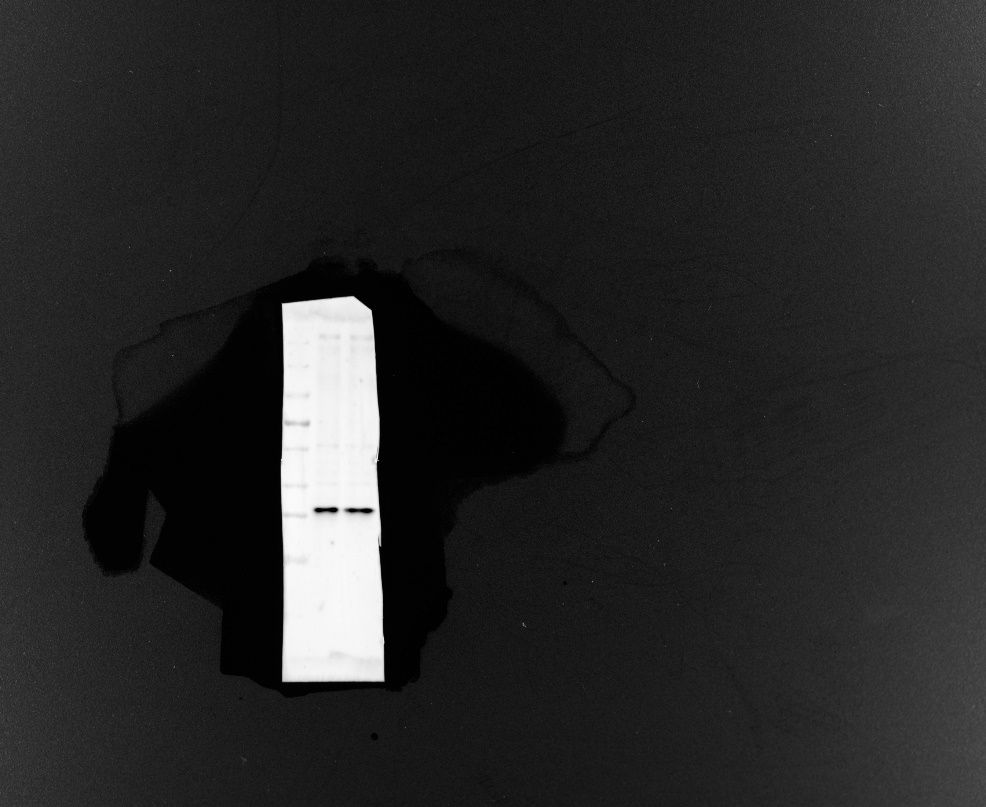


GAPDH

180 kd

135 kd

100 kd

75 kd

65 kd

45 kd

35 kd

25 kd

Supplementary figure 10. Expression of GAPDH in COS7-ACE2 cells following treatment with JHJD. Related to Figure 7A.


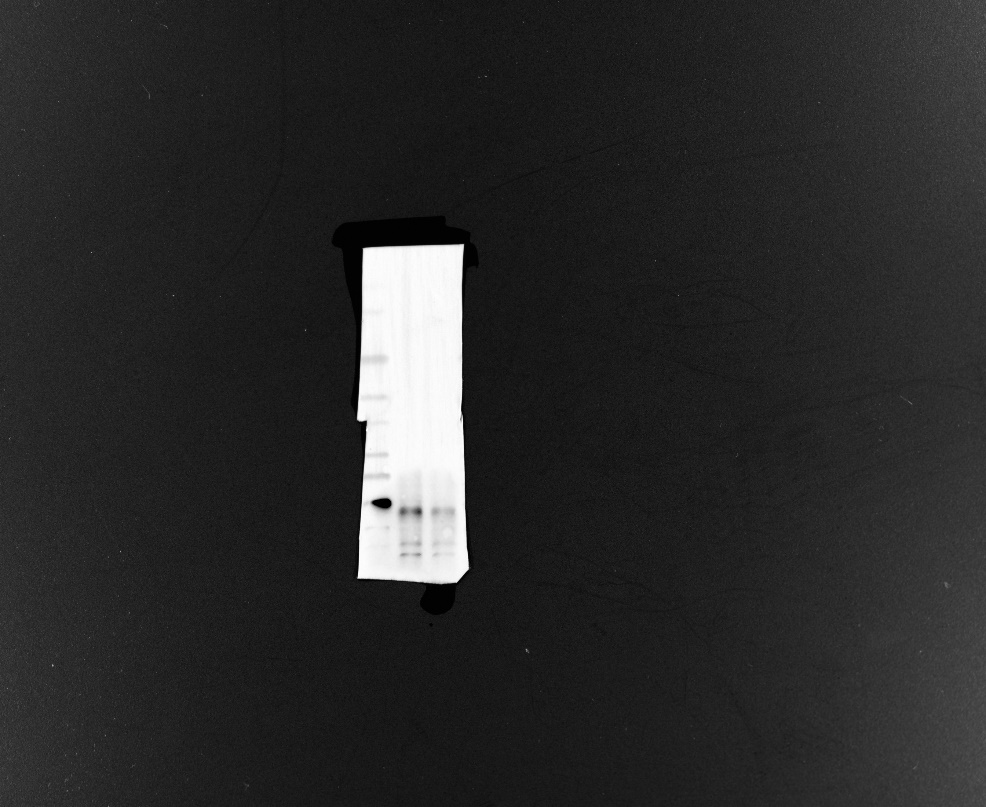


HIF-1α

180 kd

135 kd

100 kd

75 kd

65 kd

45 kd

35 kd

25 kd

15 kd

Supplementary figure 11. Expression of HIF-1α in COS7-ACE2 cells following treatment with JHJD. Related to Figure 7A.


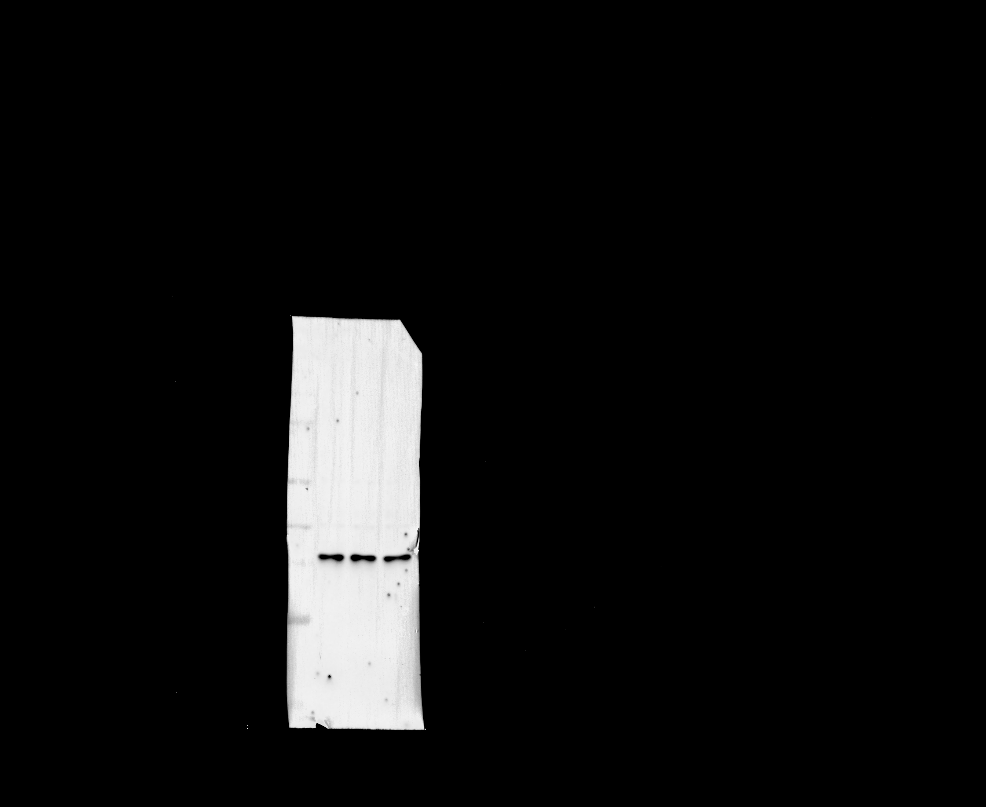


JHJD

**- - +**

**- + +**

Poly (I:C)

GAPDH

75 kd

65 kd

45 kd

35 kd

15 kd

25 kd

Supplementary figure 12. Expression of GAPDH in RAW264.7 cells following treatment with poly(I:C) and JHJD. Related to Figure 7B.


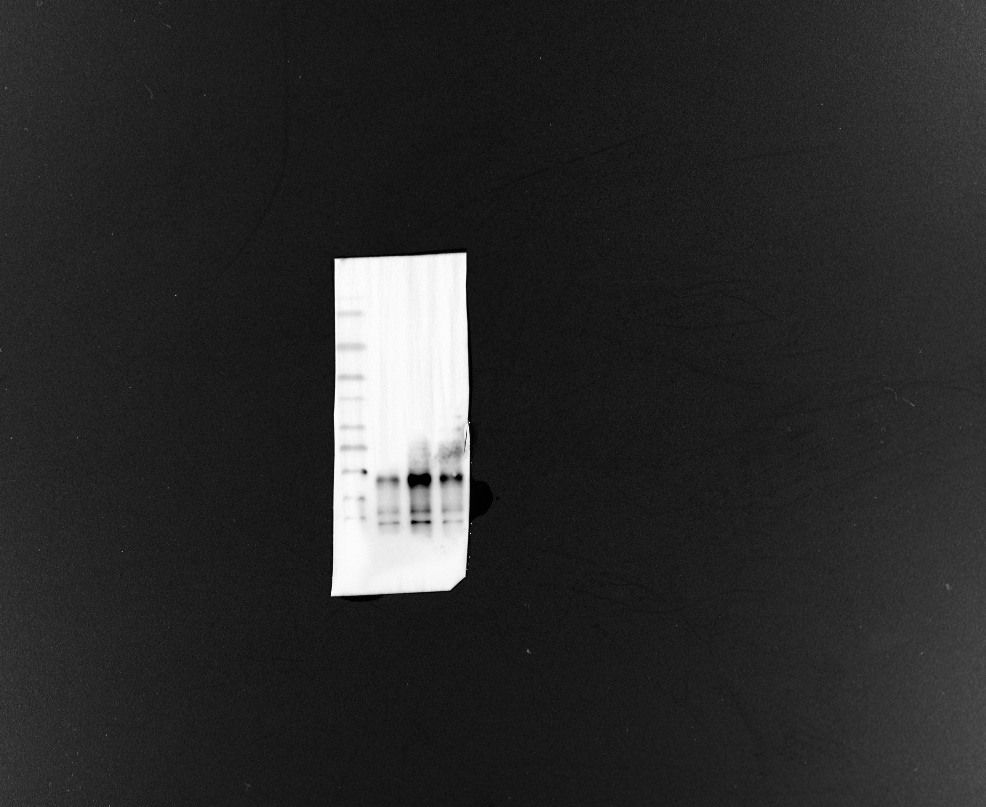


15 kd

HIF-1α

**- + +**

**- - +**

Poly (I:C)

JHJD

180 kd

135 kd

100 kd

75 kd

65 kd

45 kd

35 kd

25 kd

10 kd

Supplementary figure 13. Expression of HIF-1α in RAW264.7cells following treatment with poly(I:C) and JHJD. Related to Figure 7B.


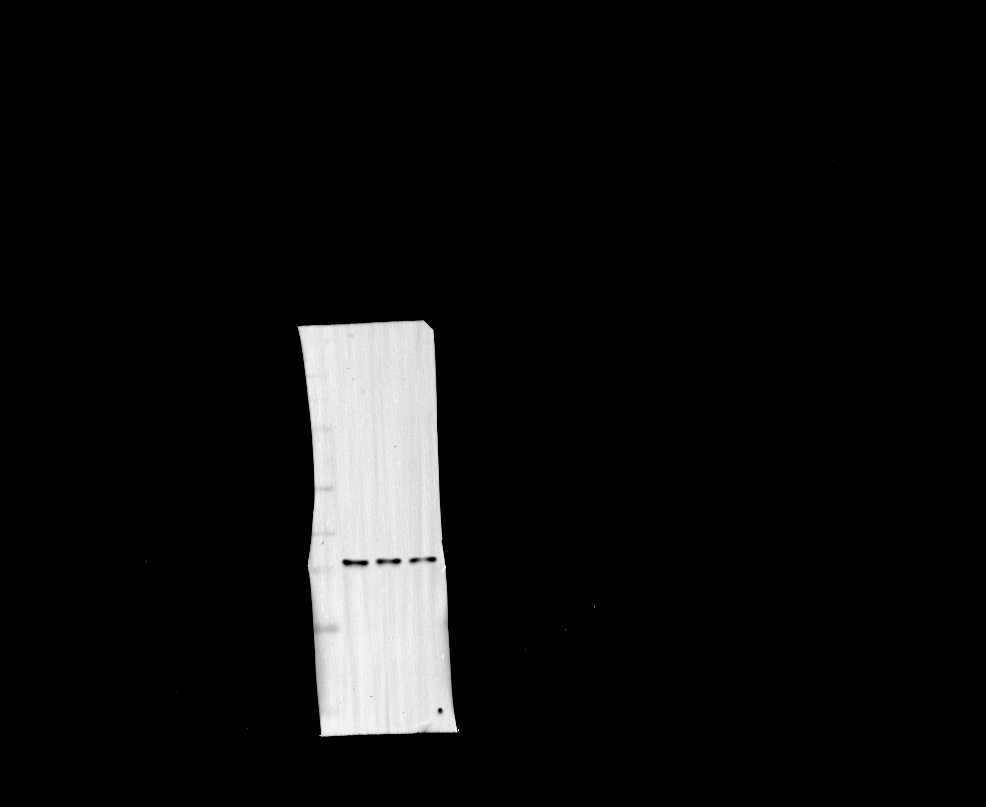


Poly (I:C)

JHJD

**- - +**

**- + +**

GAPDH

180 kd

135 kd

100 kd

75 kd

65 kd

45 kd

35 kd

25 kd

Supplementary figure 14. Expression of GAPDH in PBMC following treatment with poly(I:C) and JHJD. Related to Figure 7B.


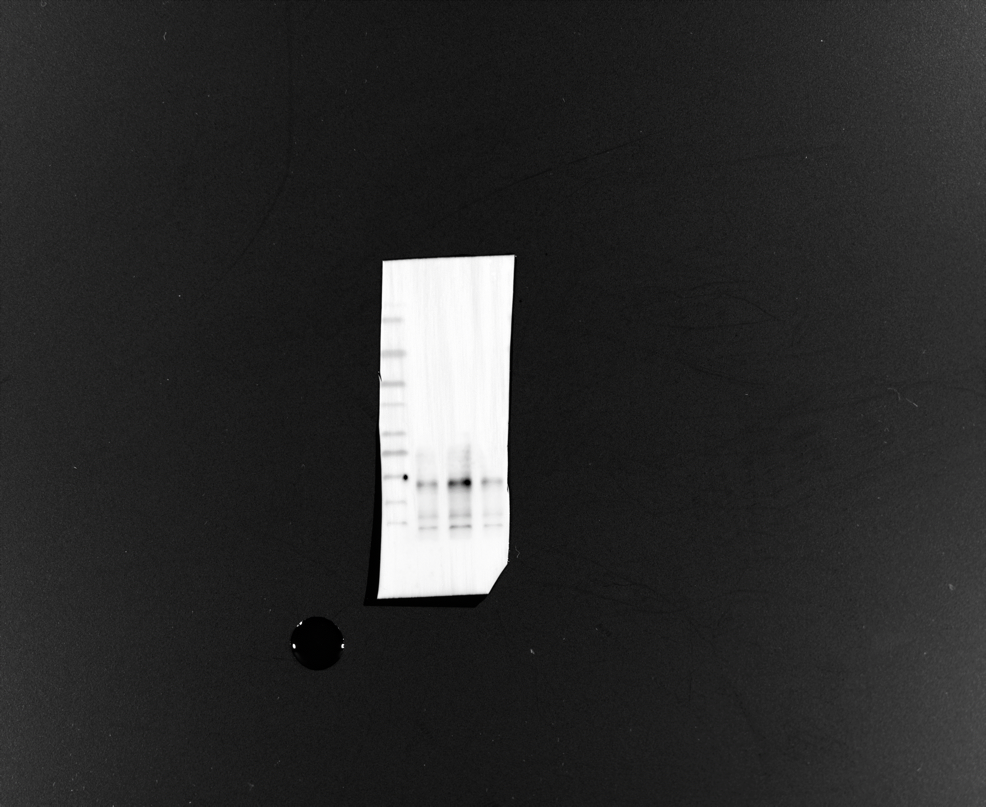


**- - +**

Poly (I:C)

JHJD

**- + +**

HIF-1α

180 kd

135 kd

100 kd

75 kd

65 kd

45 kd

35 kd

25 kd

15 kd

10 kd

Supplementary figure 15. Expression of HIF-1α in PBMC following treatment with poly(I:C) and JHJD. Related to Figure 7B.
